# Product Nkabinde Mediates Cytokine Modulation and Antiviral Activity in HIV-Infected MT4 Cells

**DOI:** 10.3390/ijms27156811

**Published:** 2026-07-29

**Authors:** Boitumelo Setlhare, Mlungisi Ngcobo, Gila Lustig, Herbert Chikafu, Nomusa Zondo, Siphathimandla Authority Nkabinde, Magugu Nkabinde, Nceba Gqaleni

**Affiliations:** 1 Discipline of Traditional Medicine, University of KwaZulu-Natal, Durban 4041, South Africa; gil.lustig@gmail.com (G.L.); nceba.gqaleni@ahri.org (N.G.); 2 Centre for Aids Programme of Research in South Africa (CAPRISA), Doris Duke Medical Research Institute, Nelson R Mandela School of Medicine, University of KwaZulu-Natal, Durban 4041, South Africa; nomusa.zondo@caprisa.org; 3 Department of Public Health Medicine, University of KwaZulu-Natal, Durban 4041, South Africa; chikafuh@gmail.com; 4 Ungangezulu, Dundee 3000, South Africamagugun@webmail.co.za (M.N.); 5 Faculty of Health Sciences, Durban University of Technology, Durban 4041, South Africa; 6 Africa Health Research Institute (AHRI), K-RITH Tower Building, Doris Duke Medical Research Institute, Nelson R Mandela School of Medicine, University of KwaZulu-Natal, Durban 4041, South Africa

**Keywords:** traditional medicine, MT4 cells, HIV infection, cytokine secretion, immunomodulation

## Abstract

During HIV infection, the immune system initiates an immune response to control the viremia. In this in vitro study, we investigated the immunomodulatory and anti-HIV potential of a traditional medicine formulation, Product Nkabinde (PN). A freeze-dried extract of PN was used to determine cytotoxicity in MT4 cells. Non-cytotoxic doses were used to evaluate the immunomodulatory and anti-HIV effects of PN using neutralization, prophylactic and treatment approaches. Post-treatment, cytokine quantification and p24 detection were performed. In the neutralization strategy, PN restored IL-1α (*p* = 0.0389) and IL-10 (*p* = 0.0443) to levels of uninfected cells. It also reduced HIV-induced elevations in IL-8 (*p* = 0.035), IP-10 (*p* = 0.0886), and MCP-1 (*p* = 0.0733) compared to HIV-infected cells. In the prophylactic approach, HIV infection upregulated IL-1α (*p* = 0.676), which was suppressed by PN. In the treatment strategy, PN reversed the elevated levels of IL-1α (*p* = 0.049). IL-10 was fully restored by PN to levels of uninfected cells. In all strategies, PN induced a significant and dose-dependent decrease in HIV replication. These findings demonstrate that PN exerts potent immunomodulatory effects all strategies used by reversing HIV-induced cytokine dysregulation, thereby inducing significant anti-HIV effects.

## 1. Introduction

During acute HIV infection, the body activates its immune system to manage the viremia. The immune response initiates the process of pathogen sensing and innate signaling by the host-infected cells [1,2]. The host has pathogen-recognition receptors (PRRs) that can sense pathogen-associated molecular patterns (PAMPS) or the viral nucleic acids, such as DNA or RNA in viral particles, and start a cell-intrinsic innate immune response that can result in antiviral defense and virus restriction [3,4]. The immune response is orchestrated by cell-mediated and soluble factors, including type I and III interferon (IFN), as well as pro-inflammatory cytokines and chemokines that recruit and activate innate immune cells, such as macrophages, natural killer cells, and dendritic cells [5,6,7]. These factors assist in controlling the viral spread and activating and modulating the adaptive immune response [8]. Cytokines and chemokines play a crucial role as hormones and neurotransmitters that mediate intercellular communication and are produced by many cell types, particularly those of the immune system [5,9]. During HIV-1 infection, CD4+ target cells are infected and can sense the pathogen and induce innate immune responses [10]. This infection progresses into a chronic phase that leads to a decrease in CD4+ T cells, and if not treated, it can lead to a weakened immune system, thus causing AIDS [11,12].

Cytokines and immune cells have been used to track the viremia and immune status of infected individuals. This makes them important biomarkers for evaluating HIV progression in people living with HIV (PLWH) [13]. Cytokines, such as some pro-inflammatory cytokines, may induce disruption of the membrane, allowing for easy access for viral particles [14]. Pro-inflammatory cytokines, such as IL-1α, IL-1β, TNF-α, IL-7 and IL-6 target T cells and macrophages, enhance HIV replication and are involved in HIV transcription [15,16]. GM-CSF targets macrophages, enhances HIV replication, and is involved in HIV transcription but inhibits virus entry [17,18]. The anti-inflammatory cytokine IL-10 targets T cells and macrophages and is involved in the suppression of pro-inflammatory cytokines synthesis and stimulates the production of B-cells for antibody production [18,19]. A study conducted by [20] demonstrated that Plectranthus barbatus extract possesses anti-inflammatory activity by reducing the production of pro-inflammatory cytokines in vitro, in addition to exhibiting antioxidant activity and HIV-1 protease inhibition.

Antiretroviral therapy has been able to control the virus and increase the life expectancy of PLWH [21,22,23]. However, the challenge with ART is the lifelong administration required for continued viral suppression in PLWH. This pill burden has led to nonadherence, relapse, and more HIV infections due to treatment interruption [24,25]. Pre-exposure prophylaxis (PrEP) was developed with emtricitabine and tenofovir disoproxil fumarate [26,27,28]. Due to the adherence problems, new PrEP strategies are being developed; one of the alternatives is a daily combination of tenofovir alafenamide/emtricitabine (TAF/FTC) [29,30]. Additionally, long-acting injectables cabotegravir (CAB-LA) and lenacapavir have been developed to improve the effectiveness of PrEP [31,32]. Microbicides (dapivirine intravaginal ring) were recommended by WHO for the prevention of HIV in women based on two phase III randomized placebo-controlled clinical trials showing a 27 and 35% relative reduction in the incidence of HIV [33]. Most of these preventative or treatments to manage HIV are not potent enough, as there are still some side effects and issues with nonadherence.

HIV treatment through immunological methods focuses on T cell therapies. Researchers developed these methods first for cancer treatment before adjusting them for HIV applications [34,35]. Antigen-expanded autologous T-cells involve extracting T cells from patients to increase their numbers outside the body, while chimeric antigen receptor (CAR) T cells receive genetic modifications to detect and eliminate HIV-infected cells. These therapeutic methods show promise but face multiple challenges, including cytokine storms and viral escape mutants, as well as T cell susceptibility to HIV and difficulties with production [36]. A different immunological approach enhances the functionality of T cells, B cells, and natural killer (NK) cells [37,38]. One therapeutic approach includes N-803 cytokine therapy along with NK cell therapy and BCL-2 inhibitors for cytopathicity enhancement and TLR inhibition [38,39]. Researchers have developed antibody-based treatments that focus on broadly neutralizing antibodies (bNabs) that target multiple HIV-1 envelope (Env) protein sites, including the V2 site and CD4 binding site, as well as the V3 glycan region [39,40]. Additionally, Dual-Affinity Re-Targeting (DART) molecules like HIVxCD3 function as bispecific antibodies to guide cytotoxic cells toward HIV-infected cells for destruction. The agents function as key elements in the “shock-and-kill” approach, which targets latent HIV reservoirs [41,42]. These interventions have shortcomings and are very expensive; therefore, there is a need to find alternative ways to combat HIV.

The study of traditional medicine (TM) is not a new field, as a significant portion of medical drugs are derived from medicinal plants or their bioactive compounds. TMs have shown the potency to reduce clinical symptoms of infectious diseases and modulate immune function [43,44]. When used in disease management, different TMs have shown the potential to target different processes and induce a cascade of anti-inflammatory cytokines to try and assist the body in fighting off infection [45]. *Euphorbia kansui (kansui)* is a plant reported to have latency reversal properties, serves as a protein kinase C agonist, and is constituted with euphols, which have anti-inflammatory properties [24]. *Plectranthus barbatus* has been shown to have anti-HIV and anti-inflammatory properties through the production of IL-10 [20]. The study aims to evaluate the in vitro immunomodulatory and anti-HIV properties of Product Nkabinde (PN), a South African traditional medicine product. Product Nkabinde (South Africa patent number 2023/03587 and Global patent number PCT/IB2024/052529) is a traditional herbal medicine formulated using a combination of four medicinal plants, namely, *Sclerocarya birrea* subsp. *afra* [Anacardiaceae] stems and leaves, *Gnidia sericocephala (Meisn.) gilg ex Engl.* [Thymelaeaceae] roots, *Senna italica* subsp. *italica* [Fabaceae] roots and *Pentanisia prunelloides (Klotzsch ex Eckl. & Zeyh.) Walp.* subsp. *prunelloides* [Rubiaceae] roots. We previously demonstrated that PN possessed in vitro immunomodulatory properties in human uninfected immune cells that may influence immune and inflammatory responses [46]. Our laboratory has also published data showing that PN possesses potent in vitro anti-HIV effects against clinical strains of HIV [47]. Furthermore, a recent molecular docking study of phytochemicals found in medicinal plants used to constitute PN revealed that the 10 hub genes regulated by this TM focus on immune regulation, metabolic modulation, viral comorbidity, carcinogenesis, and inflammation [48]. We hypothesized that PN could modulate the immune response of HIV-infected MT4 cells by altering the secretion of pro- and anti-inflammatory cytokines and concurrently exert anti-HIV effects without harming the cells. Therefore, the current study aims to build on these previous publications by evaluating the immunomodulatory and anti-HIV effects of PN in HIV-infected MT4 cells.

## 2. Results

### 2.1. Cytotoxicity

**Figure 1:**
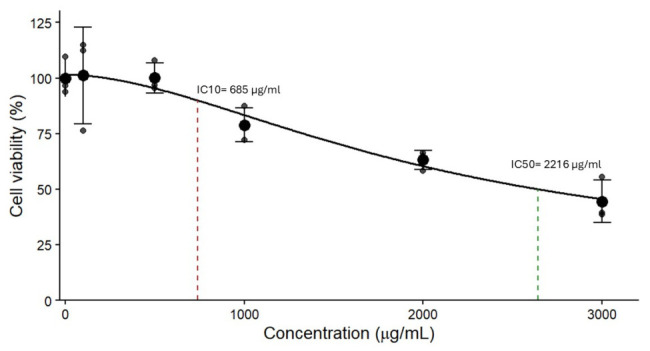
PN induced a dose-dependent cytotoxicity effect on MT4 cells over 24 h of treatment. At higher concentrations, PN was significantly cytotoxic to cells, while at lower concentrations below 1000 μg/mL, cytotoxicity was reduced. The experiment was conducted in triplicate and repeated in 2 independent experiments. Concentration–response curves were fitted using nonlinear regression with a four-parameter logistic model (variable slope) in graphpad Prism version 8. The error bars are presented as mean ± SD. The IC_10_ was 685 µg/mL (red dotted line) with a 95% CI of 4.530 to 8.093 and the IC_50_ was 2216 µg/mL (green dotted line) with a 95% CI of 2.982 to 4.907.

Product Nkabinde aqueous extract decreased the MT4 cell viability in a dose-dependent manner (Figure 1), with higher concentrations above 1000 µg/mL being significantly cytotoxic to the cells, while lower concentrations (100–1000 µg/mL) did not affect cell viability. The IC_10_ was 685 µg/mL with a 95% CI of 4.530 to 8.093, and the IC_50_ was 2216 µg/mL with a 95% CI of 2.982 to 4.907 (Figure 1).

### 2.2. Effects of Product Nkabinde in the Neutralization Treatment Strategy on Cytokines Secretion and HIV Replication

#### 2.2.1. Cytokines Secretion in the Neutralization Treatment Strategy

**Figure 2:**
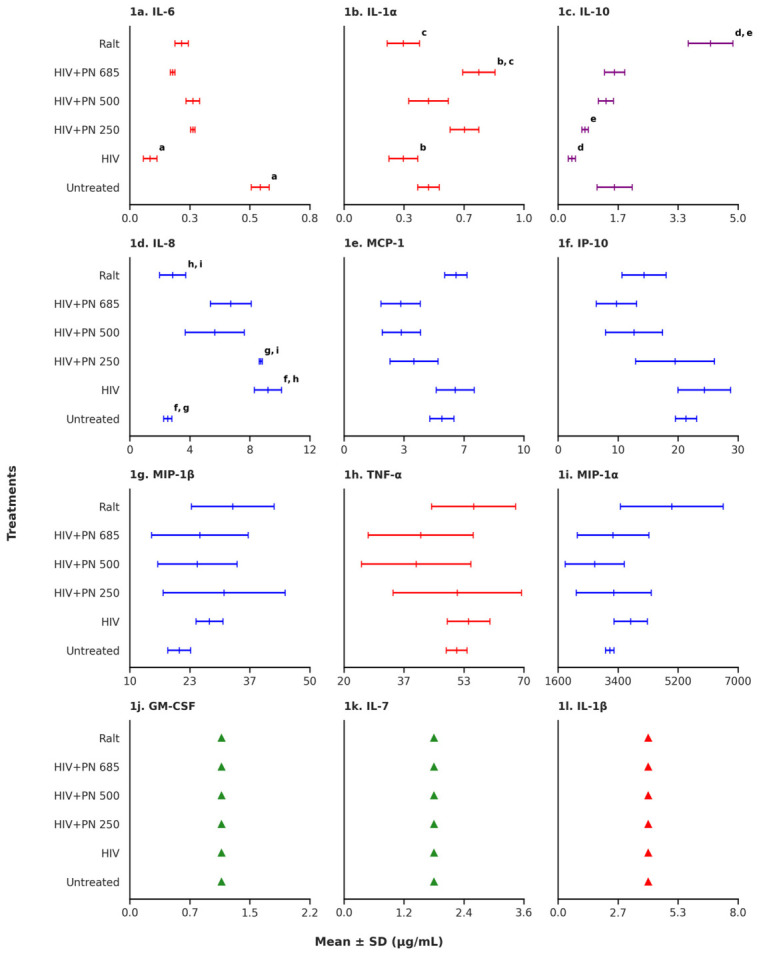
Cytokine secretion levels (mean ± SD) under various treatment conditions in the neutralization strategy. The figure illustrates individual associations between different treatment conditions and cytokine categories: pro-inflammatory (red), chemokines (blue), growth factors (green), and anti-inflammatory (purple). All the triangles represent cytokines that did not show any variations amongst treatments, and the error bars represent standard deviations. Matching lowercase superscript letters indicate statistically significant specific pairwise differences identified by post hoc tests. The non-parametric Kruskal–Wallis test was used to assess differences in cytokine distributions across the six treatment groups. Dunn’s post hoc tests with Benjamini–Hochberg false discovery rate (FDR) correction were used to identify specific pairwise differences. The experiments were conducted in triplicate and repeated in two independent experiments (N = 6). Global differences (*p* < 0.05) in cytokine distributions were detected for IL-1α, IL-6, IL-8, IL-10, IP-10, and MCP-1.

The immunomodulatory effects of PN were evaluated at the IC_10_ concentrations of 685 µg/mL and non-toxic concentrations of 500 and 250 µg/mL in a neutralization strategy (Figure 2, Tables S1, S7 and S8). The cytokine levels were quantified in HIV-infected cells and compared to untreated and raltegravir-treated HIV-infected cells. The overall analysis assessed differences across all experimental groups simultaneously and identified significant treatment effects for IL-1α (*p* = 0.0133), IL-6 (*p* = 0.0123), IL-8 (*p* = 0.0132), IL-10 (*p* = 0.0105), IP-10 (*p* = 0.0386), and MCP-1 (*p* = 0.0175), with large effect sizes (ε^2^ = 0.853, 0.863, 0.853, 0.883, 0.693, and 0.813, respectively). These results indicate that cytokine levels differ significantly across the experimental conditions. Subsequent post hoc pairwise comparisons examined specific group differences and revealed a different pattern for certain cytokines. Relative to the untreated control group, HIV infection was associated with lower levels of IL-1α (0.33 ± 0.08 vs. 0.47 ± 0.06, *p* = 0.3306) and significantly lower levels of IL-6 (0.09 ± 0.03 vs. 0.58 ± 0.04, *p* = 0.0086).

PN did not show a significant elevation of IL-1α when compared to uninfected cells (*p* > 0.05). However, it exhibited pronounced elevation in comparison to HIV infected cells (0.33 ± 0.08, *p* = 0.0389). IL-8 was markedly elevated by HIV infection when compared to unstimulated controls (9.21 ± 0.91 vs. 2.53 ± 0.28 = 0.035). Furthermore, raltegravir reduced IL-8 when compared to HIV-infected cells (9.21 ± 0.91 vs. 2.85 ± 0.87, *p* = 0.035). These findings suggest that both PN and raltegravir have a rebalancing effect on inflammatory cytokine profiles.

Anti-inflammatory IL-10 was downregulated by HIV (0.38 ± 0.1 vs. untreated 1.57 ± 0.49, *p* = 0.0997). PN restored IL-10 levels, with 685 µg/mL fully restoring secretion (1.57 ± 0.28, *p* = 1.000) to levels of the untreated control. In contrast, GM-CSF (1.13), IL-1β (4.00), and IL-7 (1.81) levels remained unchanged across all conditions, indicating these cytokines were not responsive to HIV infection or the tested treatments. MIP-1α, MIP-1β, and TNF-α exhibited variations across groups, but these differences were not statistically significant (*p* > 0.1).

#### 2.2.2. HIV Replication in the Neutralization Treatment Strategy

**Figure 3:**
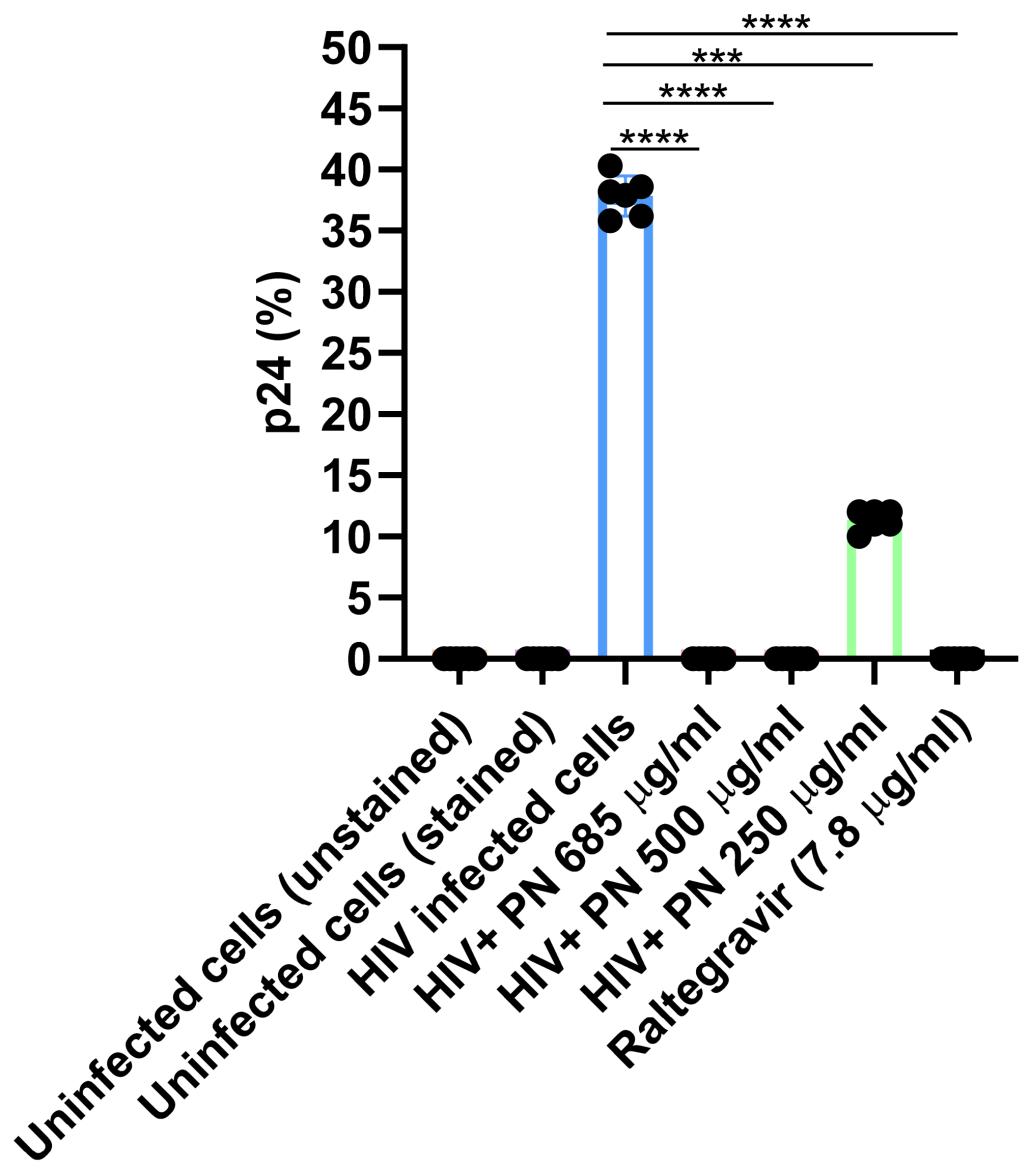
Graphic representation of the potency of PN as a neutralization agent against HIV. The three concentrations of PN (685, 500 and 250 µg/mL) were able to significantly inhibit viral replication, which was comparable to the effects of the positive control, raltegravir. The experiments were conducted in triplicates and repeated in two independent experiments (N = 6). The Tukey–Kramer multi-comparison test was used for statistical analysis, and significance is indicated as follows: *p* < 0.05 *, *p* < 0.01 **, *p* < 0.001 *** and *p* < 0.0001 ****.

Product Nkabinde showed in vitro potency as a neutralization agent against HIV (Figure 3 and Tables S2 and S9). There was approximately 37.8% detection of p24 by flow cytometry in the cells that were infected with HIV. However, in the presence of 685 µg/mL PN, there was a drastic inhibition of viral copies from 37.8 to 0% [mean 37.83; CI (36.58–39.09), *p* = 0.0001] and at 500 µg/mL from 37.8 to 0.011% [mean 37.83; CI (36.57–39.08), *p* = 0.0001]. These antiviral effects of PN were comparable to raltegravir [mean 37.83; CI (36.58 to 39.09), *p* = 0.0001]. The lowest concentration of PN used (250 µg/mL) also induced a significant decrease in p24 detected from 37.8 to 12.2% [mean 26.50; CI (25.24 to 27.76), *p* = 0.0001] when compared to the untreated control.

### 2.3. Effect of Product Nkabinde as a Prophylactic Treatment Strategy on Cytokine Secretion and HIV Replication

#### 2.3.1. Cytokines Secretion in the Prophylactic Treatment Strategy

**Figure 4:**
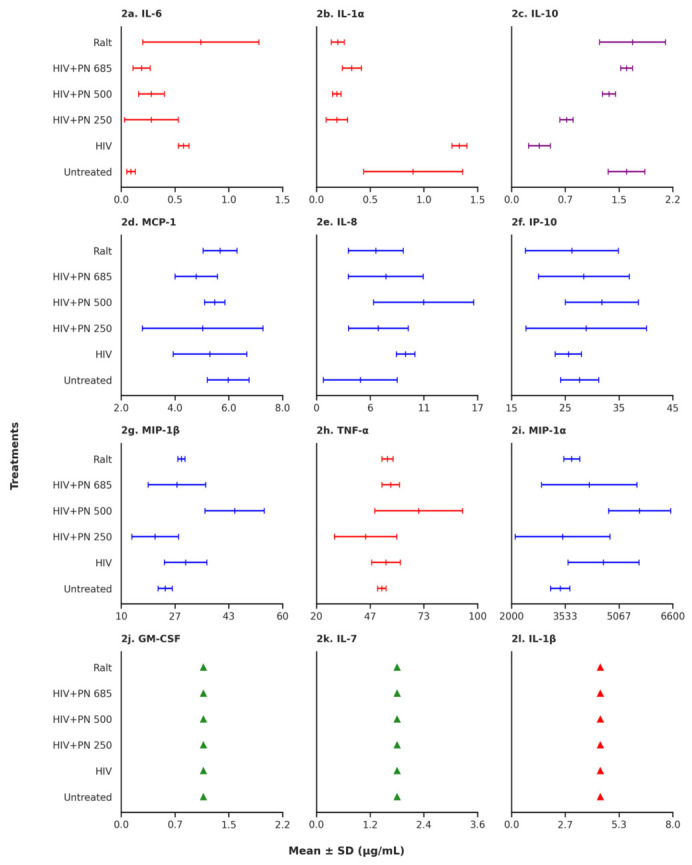
Cytokine concentration levels (mean ± SD) under various experimental conditions in the prophylactic treatment strategy. The figure illustrates individual associations between different conditions and cytokine categories: pro-inflammatory (red), chemokines (blue), growth factors (green), and anti-inflammatory (purple). All the triangles represent cytokines that did not show any variations amongst treatments. Error bars represent standard deviations. The non-parametric Kruskal–Wallis test was used to assess differences in cytokine distributions across the six treatment groups. Dunn’s post hoc tests with Benjamini–Hochberg false discovery rate (FDR) correction were used to identify specific pairwise differences. The experiments were conducted in triplicate and repeated in two independent experiments (N = 6). Although global differences were significant (*p* < 0.05) for IL-1α, IL-10, and MIP-1β, post hoc tests yielded no significant pairwise differences across any treatment groups. Consequently, no superscript significance letters are displayed for approach 2.

The overall results showed a marked increase in the secretion of IL-1α (*p* = 0.0190), IL-10 (*p* = 0.0208), and MIP-1β (*p* = 0.0394) after treatment with PN concentrations and raltegravir, with effect sizes (ε^2^) of 0.793, 0.783 and 0.693, respectively (Figure 4, Tables S3, S7 and S8). This suggests strong inhibition of HIV-induced IL-1α upregulation by both PN and the antir etroviral agent. IL-10, a key anti-inflammatory cytokine, was sharply downregulated in HIV-infected cells (0.38 ± 0.15) compared to uninfected controls (1.57 ± 0.25, *p* = 0.052). Although not statistically significant, there was a reduction in mean (SD) values of IL-10. PN treatment at 685 µg/mL fully restored IL-10 levels (1.57 ± 0.08, *p* = 0.052), with no significant effect observed at lower concentrations of PN. Raltegravir also increased IL-10 (1.65 ± 0.45, *p* = 0.052). These changes highlighted the PN’s potential to reverse HIV-mediated IL-10 suppression.

The overall differences were statistically significant and demonstrated a large effect size (*p* = 0.0394; ε^2^ = 0.693), suggesting a concentration-dependent dual effect of PN on IL-10 secretion.

HIV infection (0.58 ± 0.05) elevated the secretion of IL-6 when compared to untreated cells (0.09 ± 0.04, *p* = 0.0395). While PN reduced the levels of IL-6 at all concentrations used, the effect was not significant. Likewise, IL-8 showed increased levels following HIV infection (9.4 ± 0.98 vs. 4.64 ± 3.9), though the response to treatment with PN concentrations was not significant (*p* = 0.3853). IP-10, MCP-1, MIP-1α, and TNF-α exhibited modest changes between groups without reaching statistical significance (*p* > 0.1). Notably, TNF-α was elevated by HIV infection (54.54 ± 7.11 vs. 52.49 ± 2.13, *p* = 0.1961) when compared to uninfected untreated controls. However, it was further increased by the 500 µg/mL concentration of PN (70.75 ± 21.8), suggesting a stimulatory effect that warrants further investigation (*p* = 0.1961). No significant differences were observed in GM-CSF (1.13), IL-1β (4.00), and IL-7 (1.81), which remained stable across all conditions.

#### 2.3.2. HIV Replication in the Prophylactic Treatment Strategy

**Figure 5:**
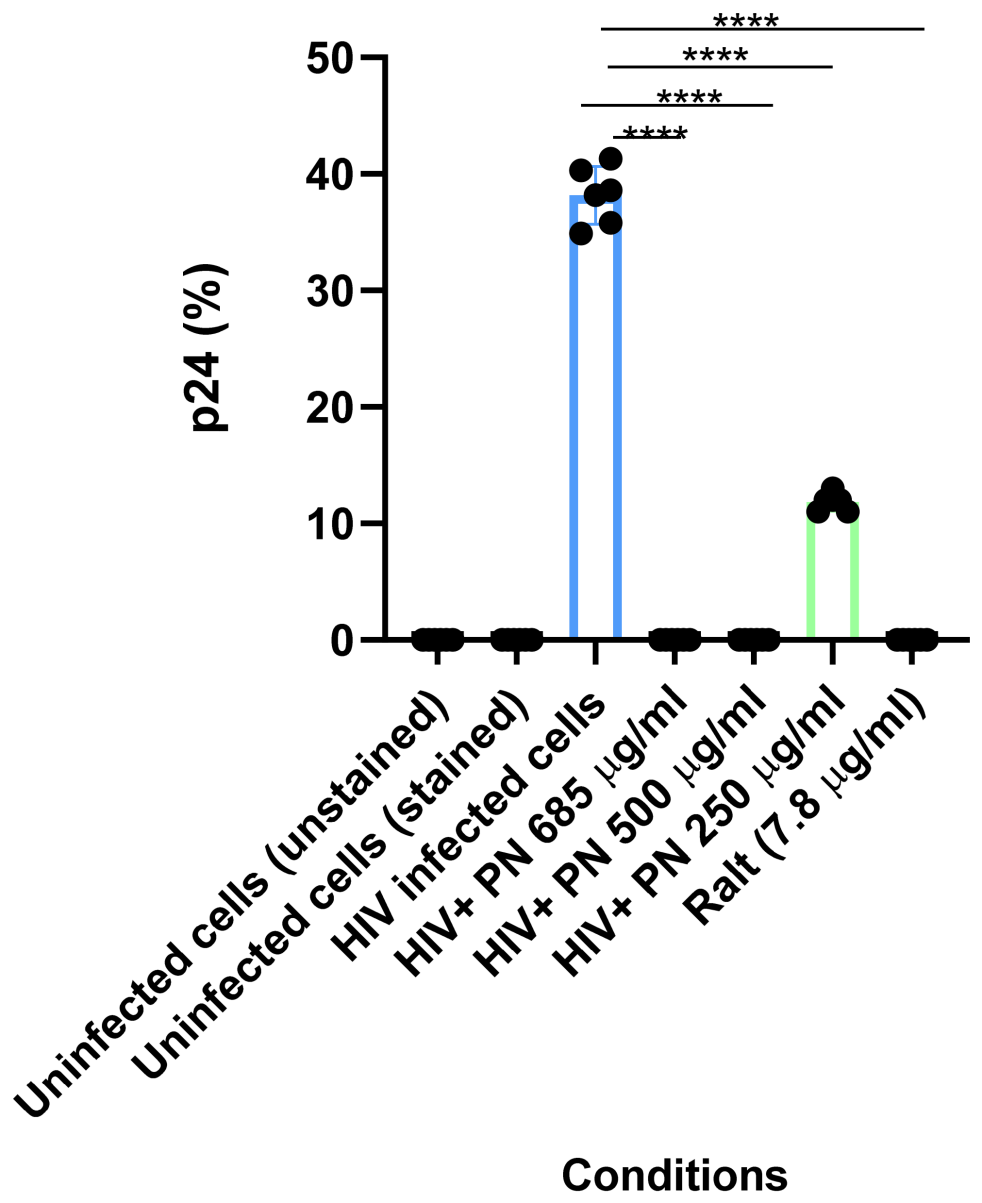
Pre-treatment of MT4 cells with PN concentrations (685, 500 and 250 µg/mL) for 8 h as a prophylactic approach, followed by HIV infection, showed comparable inhibition of viral replication to the positive control, raltegravir. The experiments were conducted in triplicate and repeated in two independent experiments (N = 6). The Tukey–Kramer multi-comparison test was used for statistical analysis, and significance is indicated as follows: *p* < 0.05 (*), *p* < 0.01 (**), *p* < 0.001 (***), and *p* < 0.0001 (****).

PN was also evaluated for its potential as a prophylactic against HIV infection (Figure 5 and Tables S4 and S9). The results showed that at concentrations of 685 µg/mL [mean 38.18; CI (36.41–39.95), *p* = 0.0001] and 500 µg/mL [mean 38.17; CI (36.40 to 39.94), *p* = 0.0001], PN inhibited HIV replication from 38.1 to 0%. This was similar to the effects of raltegravir, which significantly reduced HIV replication to 0% (*p* = 0.0001). Moreover, even at 250 µg/mL PN, there was a significant decrease in viral copies from 38.1 to 12.2% [mean 26.35; CI (24.58–28.12), *p* = 0.0001] when compared to the uninfected control. However, at this concentration, the effects of PN were not significant compared to those of raltegravir-treated cells.

### 2.4. Role of Product Nkabinde in the Post-Infection Treatment on Cytokine Secretion and HIV Replication

#### 2.4.1. Cytokine Secretion in the Post-Infection Strategy

**Figure 6:**
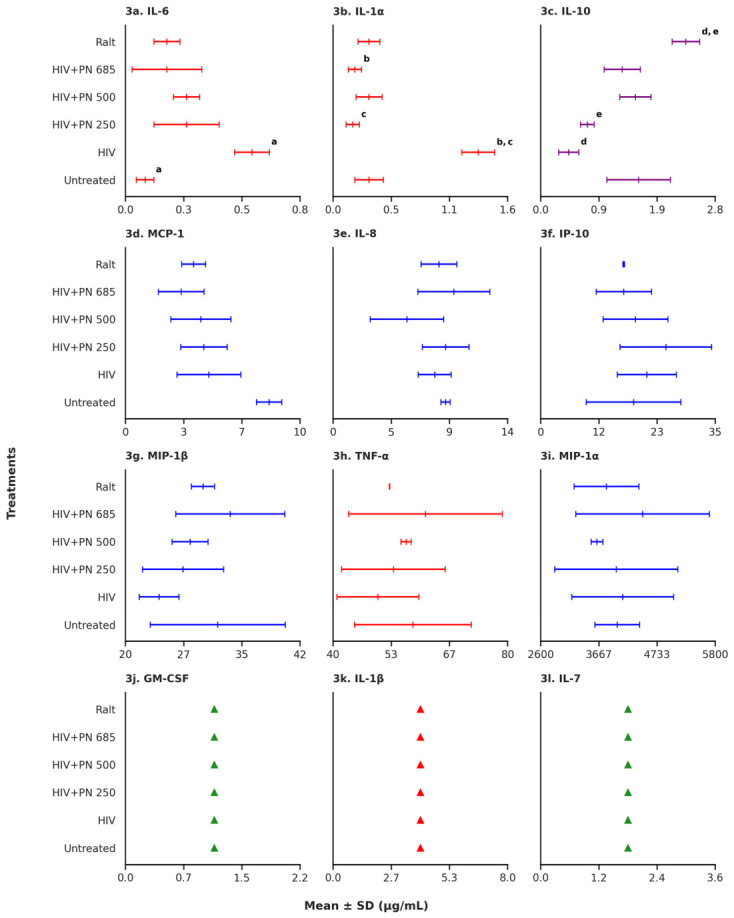
Cytokine production (mean ± SD) is induced by various concentrations (685, 500 and 250 µg/mL) of PN in the post-infection treatment strategy. The figure illustrates individual associations between different conditions and cytokine categories: pro-inflammatory (red), chemokines (blue), growth factors (green), and anti-inflammatory (purple). The non-parametric Kruskal–Wallis test was used to assess differences in cytokine distributions across the six treatment groups. Dunn’s post hoc tests with Benjamini–Hochberg false discovery rate (FDR) correction were used to identify specific pairwise differences. The experiments were conducted in triplicate and repeated in two independent experiments (N = 6). Error bars represent standard deviations. Global differences in cytokine distributions were evaluated, with significant differences (*p* < 0.05) detected for IL-1α, IL-6, and IL-10. Matching lowercase superscript letters indicate statistically significant pairwise differences identified by post hoc tests. Matching lowercase superscript letters indicate statistically significant specific pairwise differences identified by post hoc tests.

The post-infection treatment strategy demonstrated modulation of different cytokines and chemokines by PN concentrations (Figure 6, Tables S5, S7 and S8). The overall results demonstrated a pronounced elevation in IL-6 (*p* = 0.0395) and IL-1α (*p* = 0.0107), with large effect sizes, ε^2^ (0.713, 0.693 and 0.883), respectively. In the pairwise comparison, PN concentrations reversed this increase in a dose-responsive manner, reducing IL-1α significantly (0.2 ± 0.06, *p* = 0.049) at 685 µg/mL and (0.18 ± 0.06, *p* = 0.029) at 250 µg/mL. Similarly, IL-6 levels increased significantly with HIV infection (0.58 ± 0.08, *p* = 0.022) compared to uninfected controls (0.09 ± 0.04). However, with PN-treated cells, there were low levels of these cytokines, showing the anti-inflammatory properties of the TM.

A noticeable yet not significant downregulation of IL-10 was observed in HIV-infected cells (0.45 ± 0.16) compared to untreated controls (1.57 ± 0.51, *p* = 0.120). PN treatment restored, though not significantly, IL-10 levels at the higher concentrations of 685 and 500 µg/mL (1.31 ± 0.29, *p* = 0.217 and 1.52 ± 0.25, *p* = 0.121), respectively. Raltegravir led to an even greater increase (2.33 ± 0.22), *p* = 0.009) in IL-10 when compared to the untreated control. In contrast, GM-CSF (1.13), IL-1β (4.00), and IL-7 (1.81) remained unchanged across all treatment groups, showing no sensitivity to either HIV infection or subsequent treatment. Other chemokines and cytokines, including IP-10, MCP-1, MIP-1α, MIP-1β, and TNF-α, did not exhibit statistically significant differences (*p* > 0.1), although variations in secretion levels were observed. For example, HIV reduced MCP-1 levels (4.78 ± 1.83 vs. 8.23 ± 0.72 in untreated), and PN at 685 µg/mL further reduced it to 3.2 ± 1.31. However, this effect was not statistically significant (*p* = 0.2501). Similarly, MIP-1β levels decreased with HIV infection (24.25 ± 2.5 vs. 31.62 ± 8.5 for uninfected cells) but were restored by PN (33.21 ± 6.87 at 685 µg/mL), though the overall results show no significance (*p* = 0.2971). IL-8 and TNF-α levels were not significantly affected by HIV infection or treatment interventions (*p* = 0.4385 and *p* = 0.7836, respectively), despite moderate fluctuations across treatment groups.

#### 2.4.2. HIV Replication in the Post-Infection Treatment Strategy

**Figure 7:**
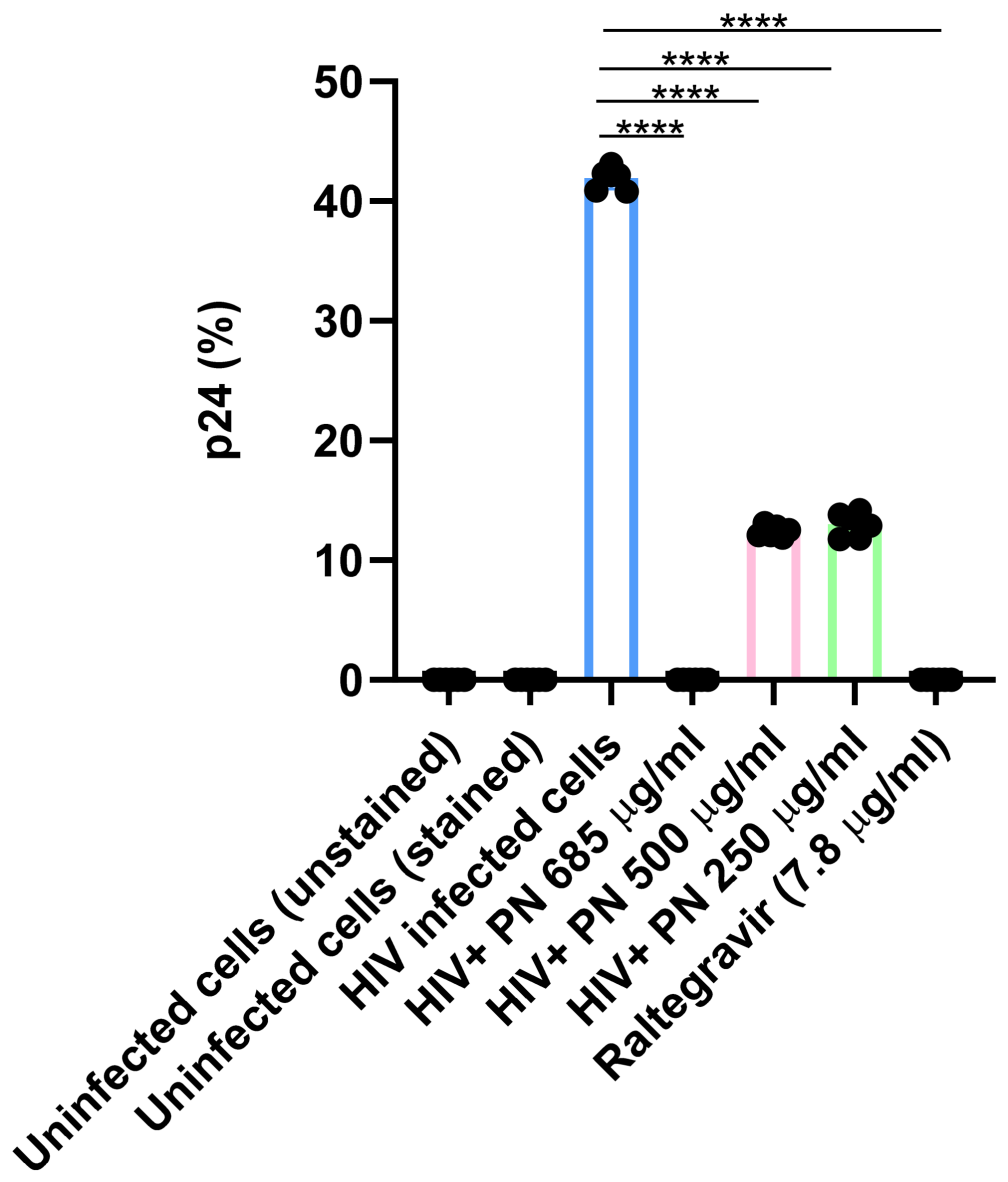
Effects of PN on HIV replication in the post-infection treatment strategy. The three concentrations (685, 500 and 250 µg/mL) of PN were able to significantly inhibit viral replication compared to untreated HIV-infected cells. As expected, raltegravir showed significant anti-HIV effects. The experiments were conducted in triplicate and repeated in two independent experiments (N = 6). The Tukey–Kramer multi-comparison test was used for statistical analysis, and significance is indicated as follows: *p* < 0.05 (*), *p* < 0.01 (**), *p* < 0.001 (***) and *p* < 0.0001 (****).

This strategy evaluated the potential of PN as a treatment for HIV infection (Figure 7 and Tables S7 and S9). The HIV p24 protein average was 41.92% after 48 h of incubation in HIV-infected MT4 cells. At the concentration of 685 µg/mL, PN inhibited HIV replication from 42.9 to 0% [mean 41.92; CI (40.93–42.90)], which was similar to the effects of raltegravir (*p* = 0.0001). PN concentrations of 500 and 250 µg/mL were able to significantly reduce viral load to 12% [mean 29.50; CI (28.52 to 30.48); *p* = 0.0001] and 14% [mean 28.92; CI 27.93–29.90); *p* = 0.0001] when compared to the HIV infected control. However, these concentrations were not significantly effective when compared to raltegravir (*p* = 0.01).

## 3. Discussion

HIV infection compromises the immune system through a direct attack on CD4+ T cells, making it weak so that the infection can progress. The immune system tries to regulate and fight the virus through various mechanisms [49]. However, as the infection intensifies and no treatment is administered, the immune system becomes overwhelmed. This is why it is important to develop both preventative and treatment strategies for HIV infection. Different approaches to prevent or treat HIV have been successful in controlling the viremia, but despite all these interventions, HIV continues to be a global health concern [50]. In this study, the MT4 cell line was infected with HIV and PN. Product Nkabinde was evaluated for its potential as an immunomodulatory and anti-HIV agent using the neutralization, prophylactic, and post-infection treatment strategies (Figures S1–S3). The MT4 cells were initially reported to be isolated from a 50-year-old Japanese person suffering from adult T-cell leukemia [51,52] and are highly susceptible and permissive to HIV infection [53,54].

Traditional medicines have been reported to have immunomodulatory properties by releasing anti-inflammatory cytokines and suppressing pro-inflammatory cytokines [55,56]. These cytokines help modulate the immune system during HIV infection to assist the immune system in warding off infections. A study by [14] showed the inhibition of HIV-1 reverse transcriptase and the anti-inflammatory properties of TMs sold in the Limpopo province of South Africa. Recently, we showed that PN modulated the immune system in healthy donors through changes in the expression of cellular receptors and cytokine secretion [46]. The current study reveals that the aqueous PN extract exhibits marked immunomodulatory activity in HIV-infected MT4 cells, significantly correcting cytokine imbalances associated with viral infection. The trend in cytokines and chemokines modulation was similar for all three strategies used in this study.

PN consistently restored IL-1α and IL-6 production to normal levels in uninfected cell samples, and this effect was comparable to that of raltegravir. IL-1α is a central mediator of innate immune activation and plays a critical role in initiating and amplifying inflammatory responses, particularly through its function as an alarmin released upon cellular damage, thereby promoting local and systemic inflammation [57]. Its modulation suggests that PN may influence early inflammatory signalling pathways that contribute to immune activation. IL-6 is a pleiotropic cytokine involved in acute-phase responses, immune cell differentiation, and chronic inflammation, and is strongly associated with persistent immune activation and disease progression in HIV infection [58]. The modulation of IL-6 in the HIV infection model further suggests that PN may exert context-dependent effects under conditions of heightened immune activation.

Interestingly, there was a pronounced elevation of IL-10 production after treatment with PN concentrations, a regulatory cytokine that modulates immune activation and protects against immune-mediated pathology. During HIV acute infection, there is suppression of IL-10, which may impair host control over inflammation [59,60,61]. PN restored IL-10 levels to similar levels as those of uninfected cells, demonstrating potent anti-inflammatory potential. Different studies have shown that metabolites isolated from plants can modulate IL-10 expression in viral infections, which results in other cells facilitating an immune response to the infections [62,63,64]. Additionally, PN reduced elevated chemokines such as IL-8, IP-10, and MCP-1, all of which are associated with HIV-induced recruitment of inflammatory cells and enhanced viral replication [65,66,67]. The downregulation of these chemokines suggests that PN cannot only limit inflammation but may also hinder viral dissemination via the chemokine axis. Although some cytokines, including GM-CSF, IL-1β, IL-7, and TNF-α, did not show differences in mean (SD) values or any significant changes, the selective modulation observed mirrors previous findings that certain plant extracts exert focused, rather than broad-spectrum immunomodulatory effects [68]. The similarity in the pattern of immunomodulation of key cytokines between PN and raltegravir further supports the in vitro potential of PN in HIV management. Taken together, these findings highlight PN’s potential to restore immune balance without causing systemic immunosuppression in vitro.

The constituents of PN have been reported to have immunomodulatory properties. The stem bark methanolic extract of *Sclerocarya birrea* inhibited the production of pro-inflammatory cytokines such as TNF-α, IL-1β, IL-6, and the subunit of pro-inflammatory cytokines IL-12 and IL-23, namely, IL-12p40. This was facilitated by suppressing NF-κB activation in macrophages stimulated with lipopolysaccharide (LPS) and infected with *Mycobacterium bovis* BCG [69]. The oil of *S. birrea* has been reported to inhibit T-cell proliferation and induce the production of anti-inflammatory cytokine IL-4 [70]. Additionally, the oil exhibits neuroprotective activity, which may be associated with its anti-inflammatory effect by regulating signalling pathways such as TNF-α and IL-1β [71]. The roots of one Gnidia species are used as an anti-inflammatory paste to treat boils [72]. *Gnidia sericocephala* has been reported to possess anti-HIV properties and to reverse latency through the activation of the PKC and NF-κB pathways. While direct cytokine assays have not been reported for the individual plant, the activation of PKC/NF κB often results in the upregulation of cytokines such as IL-2, TNF-α, and IL-6 in T cells [47,73]. Numerous metabolites are found in *Senna italica* root extracts. Resveratrol, isolated as 3,4′,5 trihydroxystilbene, has demonstrated strong antioxidant activity and downregulates pro-inflammatory cytokines, such as TNF α, IL 1β, and IL 6, and upregulates anti-inflammatory cytokines like IL 10 via SIRT1 and NF κB modulation [74,75,76]. The aqueous and methanolic extracts of *Pentanisia prunelloides* leaves and rhizomes exhibit significant anti-inflammatory and analgesic effects in rodents, as well as inhibition of COX 1 enzyme, suggesting the suppression of inflammatory mediators such as prostaglandins and potentially cytokines [77,78]. A recent molecular docking study of phytochemicals found in medicinal plants used to constitute PN revealed that the 10 hub genes regulated by this TM focus on immune regulation, metabolic modulation, viral comorbidity, carcinogenesis, and inflammation [48]. This further confirms the potential of PN as an immunomodulator with the potential to impact HIV replication.

Comparable to the observed trend in the modulation of cytokines and chemokines, the anti-HIV effects of PN were consistent across all three treatment strategies employed in this study. During HIV replication, p24 protein plays a vital role as both a structural component and a marker of HIV replication; it has gained attention in vaccine development and antiretroviral studies [79]. In cell culture, the detection of high amounts of p24 proteins using flow cytometry is an indication of high replication of HIV [80]. It has been reported that medicinal plants can target the different stages of the HIV cycle [81,82,83]. The current study utilized different strategies, including neutralization, prophylaxis, and treatment approaches to show that PN at various concentrations was able to significantly reduce the detection of p24 proteins in cells exposed to HIV. These results further confirm the observations of [47] who showed that PN had potent anti-HIV effects against four HIV clinical strains of subtype B and C.

For all treatment strategies, PN at the highest concentration used was able to eliminate the replication of the CCR5-tropic HIV (NL 4-3). This effect was comparable to raltegravir. Some TMs have shown potency to inhibit HIV [84,85]. Interestingly, PN exhibited significant anti-HIV activity at all concentrations used in the prophylactic treatment strategy. Several studies have noted that most TMs show potency as a preventative approach against HIV [86,87]. Furthermore, the constituent medicinal plants of PN have been ethnobotanically reported to treat various sexually transmitted infections (STIs). *S. birrea* has been reported to treat STIs such as *Bacterial vaginosis* and Gonorrhea [88]. Additionally, *S. birrea* has been reported to have anti-HIV properties [88,89]. *G. sericocephala* exhibits potent in vitro anti-HIV properties, including the ability to inhibit viral replication and reverse HIV latency [90,91]. *S. italica* has been reported to treat STIs such as *Candida albicans* [92,93]. The Senna genus has been reported to possess anti-HIV properties, specifically by inhibiting reverse transcriptase [94]. *P. prunelloides* roots have been reported to be effective against STIs such as *Candida albicans* [95,96].

Although the current results are groundbreaking in terms of demonstrating the in vitro immunomodulatory and anti-HIV potential of PN, the study has several limitations. The first limitation is related to the use of only one laboratory strain of HIV (NL4-3), a CCR5-tropic virus, and therefore, the current results can only be applied to similar viruses. HIV has genetic diversity, subtype variability, and resistance mutations, which make it hard to cure. Therefore, it is important to test PN against a broader range of HIV strains to determine whether its efficacy is maintained across variants. An additional limitation is that, although MT4 cells generated promising results in evaluating the in vitro immunomodulatory and antiviral effects of PN, the use of primary cells such as peripheral blood mononuclear cells (PBMCs) would have offered a more physiologically relevant model for assessing cellular activation and broader cytokines and chemokines production. It is important to note that the proposed mechanistic pathways are based on evidence from the literature and were not directly investigated in the present study. Therefore, additional molecular and virological assays are warranted to validate these findings and elucidate the molecular mechanisms underlying the biological activity of Product Nkabinde. Additionally, in vivo models are still more desirable than isolated in vitro models as they present a complex physiological immune system that is relevant for clinical assessment of PN.

In conclusion, this in vitro study demonstrated that PN can modulate the immune response in HIV-infected MT4 immune cells through changes in the production of anti-inflammatory cytokines and chemokines such as IL-10, IP-10, MCP-1, MIP-α and MIP-β, and downregulation of pro-inflammatory cytokines such IL-1α, IL-1β, and TNF-α. Additionally, PN reduced HIV replication in vitro in the neutralization, prophylactic and treatment strategies. Further studies will explore the immunomodulatory and anti-HIV effects of PN using clinical strains of HIV in vivo models, which will assist in the better understanding of PN as a therapeutic agent.

## 4. Materials and Methods

### 4.1. Collection and Identification of Plant Samples

Plant samples used to constitute PN were collected in accordance with good collection practices [97]. PN is formulated using four medicinal plant parts, namely *Sclerocarya birrea* subsp. afra [Anacardiaceae], *Gnidia sericocephala (Meisn.) gilg ex Engl.* [Thymelaeaceae], *Senna italica subsp. italica* [Fabaceae], and *Pentanisia prunelloides (Klotzsch ex Eckl. & Zeyh.) Walp.* subsp. *prunelloides* [Rubiaceae]. All these medicinal plants were collected from Tugela Ferry in Msinga Local Municipality (GPS coordinates −28.46844, 30.47096) and delivered by a Traditional Health Practitioner (THP), Mr. Nkabinde, who disclosed the traditional uses of the plants to the research team. The plants were verified and classified by Ms. Magda Nel, an ethnobotanist at the H.G.W.J. Schweickerdt Plant Herbarium at the University of Pretoria, where specimens were deposited, as shown in Table 1. Product Nkabinde was prepared according to the instructions of the THP. Briefly, the plant material from the four individual medicinal plants was dried and ground into powder using a pestle and mortar. The resultant powder was combined at a 1:1:1:1 ratio and boiled in water for 5–8 min, followed by cooling at room temperature. The extract was then filter-sterilized and freeze-dried after 48 h of freezing using a Benchtop Freeze Dryer (VirTis SP Scientific, Warminster, PA, USA).

This is a Type C extract consisting of botanical drugs and their extracts derived from lesser-studied species and the drugs derived from them, which are not included in national or regional pharmacopeia and are not used commercially at an international level [98]. A patent application has been submitted in South Africa under reference number 2023/03587, and a global patent application has been filed under reference number PCT/IB2024/052529 for PN. The metabolites analysis of PN (Table 2) using ultra-high-performance liquid chromatography-high resolution mass spectrometry (UPLC-HRMS) is fully described in our previous publication [46].

### 4.2. Cell Culture and Treatment

Dr. Gila Lustig and Mrs. Yashica Ganga provided a frozen stock of Human T cell leukemia MT4 cell line from the Africa Health Research Institute (AHRI), University of KwaZulu-Natal, Durban. The cryovial was resuscitated in Roswell Park Memorial Institute (RPMI) (Thermofisher Scientific, Johannesburg, South Africa), media supplemented with 10% heat-inactivated fetal calf serum (FCS) (HyClone, Gauteng, South Africa), non-essential amino acids (NEAA) (Thermofisher Scientific), sodium pyruvate (Na-pyruvate) (Thermofisher Scientific), 2-(4-(2-Hydroxyethyl)-1-piperazinyl) ethanesulfonic acid (Hepes) (Thermofisher Scientific, South Africa), and L-glutamine (L-glut) (Thermofisher Scientific) in T-75 cell culture flasks. After the cells reached confluence, 5.5 × 10^5^ cells of these cells were plated in different wells of a 24-well plate at a final volume of 1 mL. These cells were then treated with different concentrations of PN ranging from 100 to 3000 µg/mL. The cells were incubated for 48 h at 37 °C, 5% CO_2_, and 95% humidity.

### 4.3. Cytotoxicity Evaluation Using the ATP Assay

At the end of the incubation period, the CellTiter-GloTM reagent (Promega, Madison, WI, USA) was added to each well according to the manufacturer’s protocol, followed by an additional 10-min incubation in the dark at room temperature. The relative light units (RLU) of the samples in each well were measured using a Glo-max luminometer (Promega, Multi-detection system) following the manufacturer’s instructions. A dose–response curve was generated for the ATP levels using the RLU and the dilutions of PN and different control samples. The 10 and 50% inhibitory concentrations (IC_10_ and IC_50_) of PN were calculated from the dose-response curve.

### 4.4. Virus Generation

Stocks of the HIV-1 NL4-3 were donated by Dr Gil Lustig from AHRI, University of KwaZulu-Natal, Durban. The virus stocks were prepared by transfection of HEK-293T cells with cloned proviral DNA. HIV-1-based lentiviruses carrying reporter genes were made by co-transfection of DNA constructs as follows: 2 × 10^6^. 293T cells were co-transfected with 10 µg of pNL-GFP-RRE-SA, 7.5 μg of pCMV-R8.2, and 2.5 μg of pMD.G using the calcium phosphate method (Promega). Viruses were harvested 2 days after co-transfection, filtered through a 0.45 μm filter, and stored at −80 °C until use.

### 4.5. Raltegravir Formulation

A powdered sample of raltegravir (Ralt—10 mg) (Merck Life Science, Modderfontein, South Africa) was used as a positive control and dissolved in 10 µL of DMSO, followed by vortexing and then further diluted in 990 µL of phosphate-buffered saline (PBS) to make a stock solution of 10 mg/mL. To establish the IC_10_ dose, titration studies to evaluate the cytotoxicity of raltegravir on MT4 cells were undertaken. The 7.8 μg/mL dose was shown to be the lowest dose that had 90% cell viability (IC_10_) and was used for further efficacy studies.

### 4.6. Strategy 1: Impact of Product Nkabinde on Neutralization Strategy

**Figure 8:**
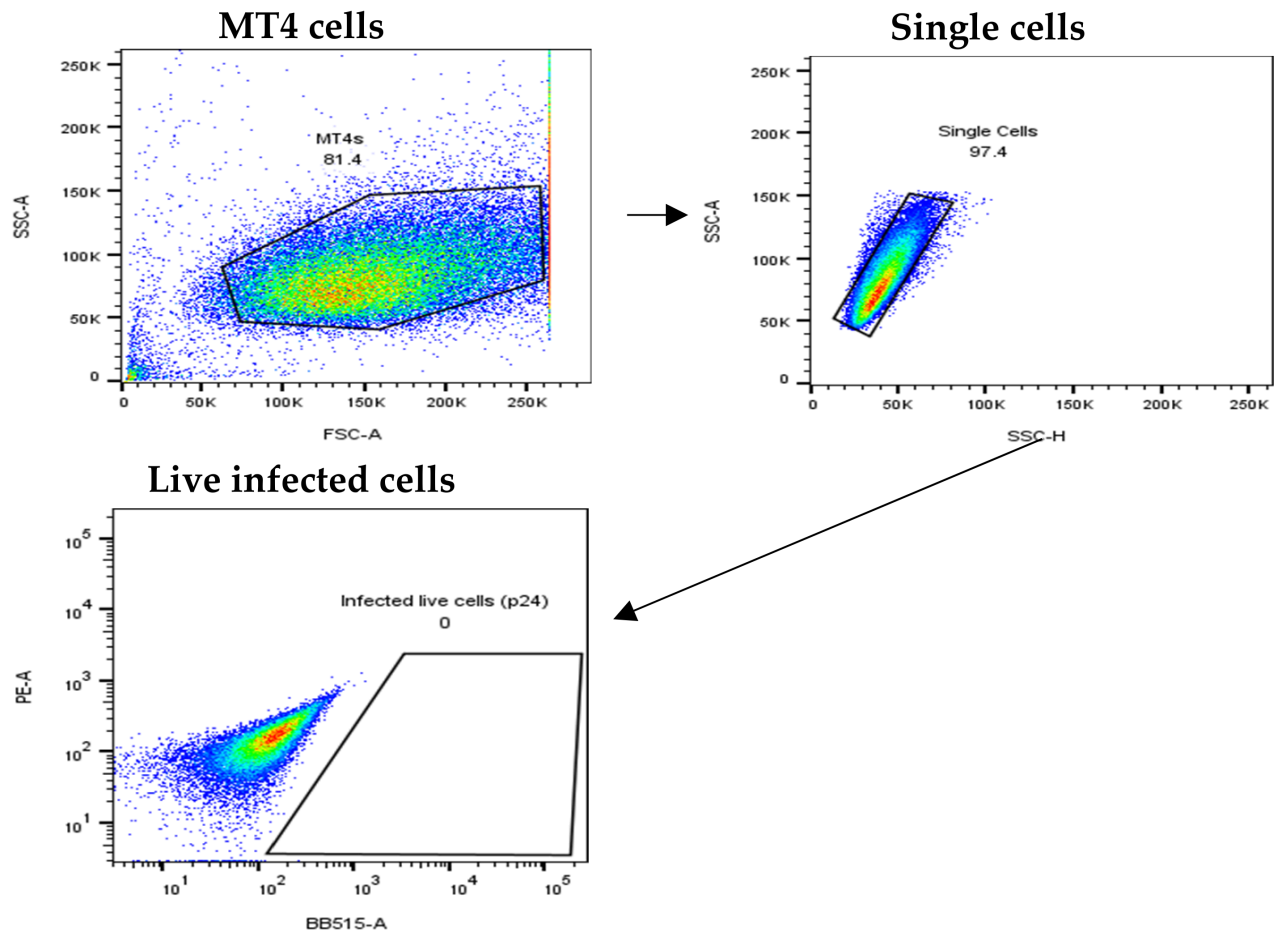
Sequential gating approach used for MT4 cells, including FSC/SSC cell selection, singlet discrimination, and identification of p24-positive cells.

Product Nkabinde at concentrations ranging from IC_10_ (685 µg/mL) and below (500 and 250 µg/mL), raltegravir (7.8 µg/mL) and HIV NL4-3 laboratory strain (1:10, 2 × 10^8^ viral RNA copies) were added into wells of a 96-well plate and incubated for an hour. After an hour, 5.5 × 10^5^/mL of MT4 cells were added and incubated at 37 °C, 5% CO_2_ for 48 h. After 48 h post-infection, the infected cells were centrifuged, and the supernatants were stored at −80 °C for cytokines quantification. The cells were used to determine the number of infected cells by fixing and permeabilizing treated MT4 cells using the BD Cytofix/Cytoperm Fixation/Permeabilization kit (BD Biosciences) according to the manufacturer’s instructions. Cell permeabilization was achieved by washing the cells with PBS containing 1% FCS (HyClone) and were then stained with anti-HIV p24 FITC conjugated antibody (KC57-FITC, Beckman Coulter, Brea, CA, USA) to detect the presence of intracellular HIV Gag protein by FACSymphony™ A5 (BD Biosciences, Johannesburg, South Africa) flow cytometry; the BB515 was the channel used to read the p24 FITC, and data were analyzed using Flowjo v10.10.0 software. The gating strategy showing FSC/SSC cell selection, singlet discrimination, and identification of p24-positive cells is in (Figure 8, Tables S2, S4 and S6). HIV-infected cells and HIV-infected raltegravir-treated cells served as negative and positive controls, respectively.

### 4.7. Strategy 2: Prophylactic Effect of Product Nkabinde on HIV Infected Cells

Product Nkabinde at concentrations ranging from IC_10_ (685 µg/mL) and below (500 and 250 µg/mL), together with raltegravir (7.8 µg/mL), were added into 5.5 × 10^5^/mL MT4 cells for 8 h, followed by the addition of 1:10 (2 × 10^8^ viral RNA copies) of HIV NL4-3 laboratory strain to infect the treated cells and incubated at 37 °C, 5% CO_2_ for 48 h. At the end of the incubation period, the infected cells were centrifuged, and supernatants were stored at −80 °C for cytokine quantification. The number of infected cells was determined by fixing and permeabilizing MT4 cells using the BD Cytofix/Cytoperm Fixation/Permeabilization kit (BD Biosciences) according to the manufacturer’s instructions. Cells were permeabilized with PBS containing 1% FCS (HyClone) and then stained with anti-HIV p24 FITC conjugated antibody (KC57-FITC, Beckman Coulter, Brea, CA, USA) to detect the presence of intracellular HIV Gag protein by FACSymphony™ A5 (BD Biosciences) flow cytometry; the BB515 was the channel used to read the p24 FITC, and data were analyzed using Flowjo v10.10.0 software. The gating strategy showing including FSC/SSC cell selection, singlet discrimination, and identification of p24-positive cells is in (Figure 8, Tables S2, S4 and S6). HIV-infected untreated cells and HIV-infected raltegravir treated cells served again as negative and positive controls, respectively.

### 4.8. Strategy 3: Effect of Post-HIV Infection Treatment with Product Nkabinde

HIV NL4-3 laboratory strain (1:10, 2 × 10^8^ viral RNA copies) was used to infect 5.5 × 10^5^/mL MT4 cells for 8 h in different wells of a 96-well plate, followed by treatment with various concentrations of PN ranging from IC_10_ (685 µg/mL) and below (500 and 250 µg/mL), as well as raltegravir (7.8 µg/mL). This was followed by incubation at 37 °C, 5% CO2 for 48 h. At the end of the incubation period, the infected cells were centrifuged, and the supernatant was stored at −80 °C for cytokine quantification. The number of infected cells was determined by fixing and permeabilizing MT4s using the BD Cytofix/Cytoperm Fixation/Permeabilization kit (BD Biosciences) according to the manufacturer’s instructions. Cells were permeabilized with PBS containing 1% FCS (HyClone), then stained with anti-HIV p24 FITC conjugated antibody (KC57-FITC, Beckman Coulter, Brea, CA, USA) to detect the presence of intracellular HIV Gag protein using FACSymphony™ A5 (BD Biosciences) flow cytometry; the BB515 was the channel used to read the p24 FITC, and data were analyzed using Flowjo v10.10.0 software. The gating strategy showing FSC/SSC cell selection, singlet discrimination, and identification of p24-positive cells is in (Figure 8, Tables S2, S4 and S6). HIV-infected untreated cells and HIV-infected raltegravir treated cells served again as negative and positive controls, respectively.

### 4.9. Cytokine Quantification

The concentrations of 12 cytokines were assessed from stored undiluted cell supernatants using the MILLIPLEX^®^ MAP human cytokine/chemokine magnetic bead panel (Merck KGaA, Darmstadt, Germany) as per the manufacturer’s instructions. The cytokines panel included pro-inflammatory cytokines: IL-1α, IL-β, IL-6, TNF–α, GM–CSF; chemokines (interleukin (IL)-8, interferon gamma-induced protein (IP)-10, monocyte chemotactic protein (MCP)-1, macrophage inflammatory protein (MIP)–1α and macrophage inflammatory protein (MIP)-1β), as well as hematopoietic cytokines (IL-7) and regulatory cytokines (IL-10). Data were acquired on the Bio-Plex^®^ (Luminex) 200 Multiplexing Immunoassay System (Bio-Rad Laboratories, Hercules, CA, USA). Optimization of standard curves was performed automatically using the Bio-Plex^®^ Manager software version 6.1 (Bio-Rad Laboratories, Hercules, CA, USA). Values with coefficients of variation (%CV) <20% and with observed recoveries between 70 and 130% were considered reliable. Values that were below the detectable limit were assigned to half of the lowest limit of detection value (LLOD), while values that were above the detectable limit were assigned double the highest limit of detection (HLOD) value.

### 4.10. Statistical Analysis

The IC_10_ and IC_50_ of PN were determined using a dose–response curve in GraphPad Prism 8 (GraphPad, San Diego, CA). The study evaluated the cytokine concentrations using mean ± standard deviation (SD) and median (interquartile range, IQR), Table S2, whereas the mean (SD) was calculated from triplicate observations. In two independent experiments that were treatment-specific, each cytokine’s median (IQR) was calculated from the treatment-level means to summarize each cytokine for the six treatments. The descriptive statistics were presented for all treatment approaches. Given the small sample size (n = 6 per cytokine per treatment), the non-parametric Kruskal–Wallis test was used to assess differences in cytokine distributions across the six treatment groups. To evaluate the practical importance of any observed difference, epsilon-squared (ε^2^) effect size was calculated, with the following thresholds: small (<0.06), medium (0.06–0.14), and large (≥0.14). Where the Kruskal–Wallis test was significant (*p* < 0.05), we conducted Dunn’s post hoc tests with Benjamini–Hochberg false discovery rate (FDR) correction to identify specific pairwise differences. Cytokines showing adjusted *p*-values ≤ 0.05 were considered significantly different between treatment groups. However, cytokines with identical concentrations across all treatment groups (GM-CSF, IL-1β, IL-7) were included in descriptive summaries but excluded from inferential analyses due to the absence of variation. The experiments were done in triplicate and repeated in two independent experiments for the anti-HIV studies. Data analysis was conducted on GraphPad Prism 8 (GraphPad, San Diego, CA) to obtain descriptive statistics (mean (SD), Median, and IQR). The different levels of significance within the separate treated groups were analyzed using one-way analysis of variance (ANOVA), and the differences between the HIV-infected cells and HIV-infected and treated cells and the control cells were analyzed using the Tukey–Kramer multiple comparison test. Differences with *p* < 0.05 were considered statistically significant. All statistical analyses were performed using GraphPad Prism 8.0 (GraphPad Inc., La Jolla, CA, USA) and R software (RStudio v 2025.5.0.496).

## 5. Conclusions

In conclusion, this in vitro study demonstrated that PN can modulate the immune response in HIV-infected MT-4 immune cells through changes in the production of anti-inflammatory cytokines and chemokines such as IL-10, IP-10, MCP-1, MIP-α and MIP-β, and downregulation of pro-inflammatory cytokines such IL-1α, IL-1β, and TNF-α. Additionally, PN reduced HIV replication in vitro in the neutralization, prophylactic and treatment strategies. Taken together, these findings provide a foundation for future molecular and virological investigations aimed at validating the proposed mechanisms and further elucidating the biological activity of Product Nkabinde. Further studies should explore the immunomodulatory and anti-HIV effects of PN using clinical strains of HIV in vivo models, which will assist in the better understanding of PN as a therapeutic agent.

## 6. Patents

A patent application for PN has been submitted in South Africa with reference number 2023/03587 and a global patent with reference number PCT/IB2024/052529 has also been lodged.

## Figures and Tables

**Table 1:** Botanical identification of the medicinal plants collected to constitute Product Nkabinde, and their voucher specimen numbers at the H.G.W.J Schweickerdt Plant Herbarium at the University of Pretoria.

Plant Name	Plant Part	Voucher Number
*Sclerocarya birrea*	Stems and leaves	126589
*Gnidia sericocephala*	Roots	126590
*Senna italica*	Roots	126591
*Pentanisia prunelloides*	Roots	126592

**Table 2:** Major bioactive compounds of Product Nkabinde are daphnane diterpenoids.

Name of Compound
Yuanhuacine A
Gniditrin
Yuanhuajine
Yuanhuacine

## Data Availability

The original contributions presented in this study are included in the article/Supplementary Materials. Further inquiries can be directed to the corresponding authors.

## Supplementary Materials

The following supporting information can be downloaded at: https://www.mdpi.com/article/10.3390/ijms27156811/s1.

## Abbreviations

The following abbreviations are used in this manuscript:

PRRs	Pathogen-recognition receptors
PAMPS	Pathogen-associated molecular patterns
IFN	Interferon
PLWH	People living with HIV
IL	Interleukin
GM-CSF	Granulocyte macrophage colony-stimulating factor
ART	Antiretroviral treatment
PrEP	Pre-exposure prophylaxis
CAB-LA	Cabotegravir
WHO	World Health Organization
CAR	Chimeric antigen receptor
NK	Natural killer
bNabs	Broadly neutralizing antibodies
DART	Dual-Affinity Re-Targeting
TM	Traditional medicine
PN	Product Nkabinde
PBMCs	Peripheral blood mononuclear cells
IC_10_	10% Inhibitory concentration
IC_50_	Half Maximum concentration
Ralt	Raltegravir
MIP	Macrophage inflammatory protein
IP-10	Interferon gamma-induced protein
MCP-1	Monocyte chemotactic protein
