# Supplementary material for: Product Nkabinde Mediates Cytokine Modulation and Antiviral Activity in HIV-Infected MT4 Cells
Source: Int J Mol Sci. 2026 Jul 29;27(15):6811. doi: 10.3390/ijms27156811 (PMC13467175; doi:10.3390/ijms27156811)
Supplement: Supplementary file 1 [file ijms-27-06811-s001.zip › ijms-4325606-supplementary.pdf]

Supplementary Data S1

Table S1. Post hoc pairwise comparisons (FDR-adjusted *p*-values) of cytokines and chemokines secretion that were significantly changed by different treatments in the neutralization treatment strategy. The experiments were conducted in triplicate and repeated in two independent experiments (N = 6). Statistical significance is indicated by asterisks where $p < 0.05$ *, $p < 0.01$ **, and $p < 0.001$ ***.

Treatments	IL-1α	IL-6	IL-8	IL-10	IP-10	MCP-1
Uninfected vs. HIV	0.3306	0.0086 *	0.035 *	0.0997	0.7458	0.714
Uninfected vs. HIV + PN685	0.2910	0.0622	0.3015	1.0000	0.1613	0.1351
Uninfected vs. HIV + PN500	0.9085	0.4261	0.3015	0.6925	0.2312	0.1351
Uninfected vs. HIV + PN250	0.3334	0.2943	0.035 *	0.2365	0.814	0.3219
Uninfected vs. Ralt	0.3306	0.2308	1.0000	0.3317	0.3134	0.714
HIV vs. HIV + PN685	0.0389 *	0.5272	0.3015	0.0997	0.0886	0.0733
HIV vs. HIV + PN500	0.3334	0.0885	0.3015	0.2312	0.1613	0.0733
HIV vs. HIV + PN250	0.0659	0.1598	0.9412	0.6141	0.6061	0.1351
HIV vs. Ralt	0.9085	0.2308	0.035 *	0.0087 *	0.1677	0.939
HIV + PN685 vs. HIV + PN 500	0.276	0.2502	0.9412	0.6925	0.6759	0.939
HIV + PN685 vs. HIV + PN 250	0.7772	0.3681	0.3142	0.2365	0.1677	0.714
HIV + PN685 vs. Ralt	0.0389 *	0.5167	0.3015	0.3317	0.6004	0.0733
HIV + PN500 vs. HIV + PN250	0.3306	0.7306	0.3015	0.3877	0.3163	0.714
HIV + PN500 vs. Ralt	0.3306	0.5666	0.3015	0.2312	0.8185	0.0733
HIV + PN250 vs. Ralt	0.0574	0.7306	0.035 *	0.0443 *	0.4189	0.1351

Table S2. Effect of PN on HIV replication using the neutralization treatment strategy. Uninfected, unstained, and uninfected stained cells served as the negative controls. Infected, stained, and untreated cells and raltegravir-treated infected cells served as the experimental samples.

<table>
<tr><th>Sample</th><th>MT4 Cells</th><th>Single Cells</th><th>Live Infected Cells</th></tr>
<tr><td>Uninfected (Unstained)</td><td>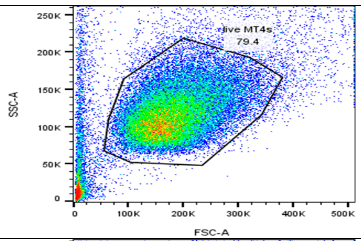
</td><td>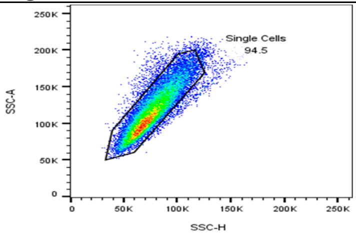
</td><td>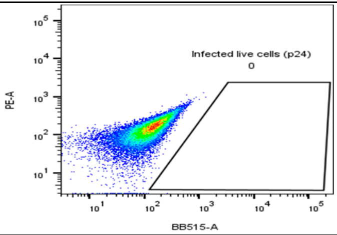
</td></tr>
<tr><td>Uninfected (stained)</td><td>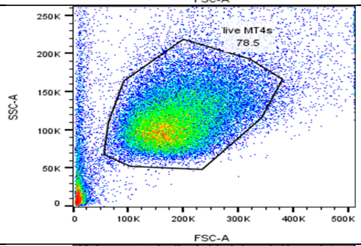
</td><td>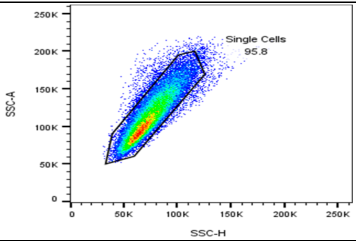
</td><td>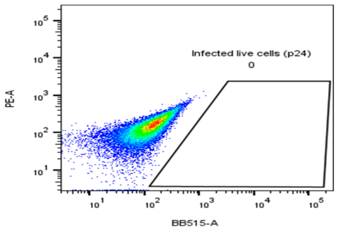
</td></tr>
<tr><td>HIV (1:10)</td><td>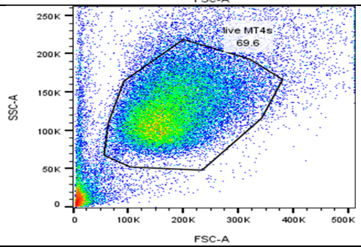
</td><td>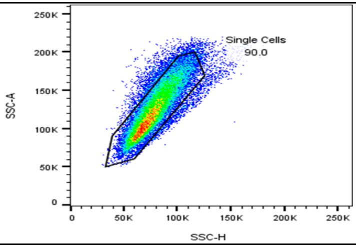
</td><td>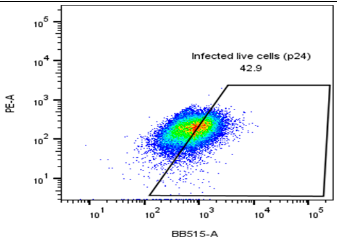
</td></tr>
</table>

Raltergravir (7.8 µg/mL)

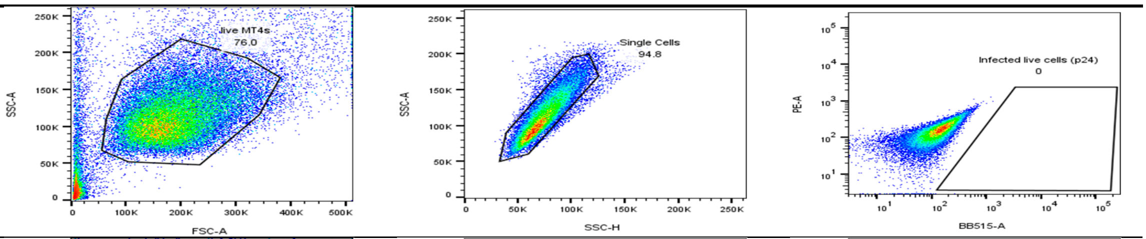

HIV (1:10) + PN 685 µg/mL

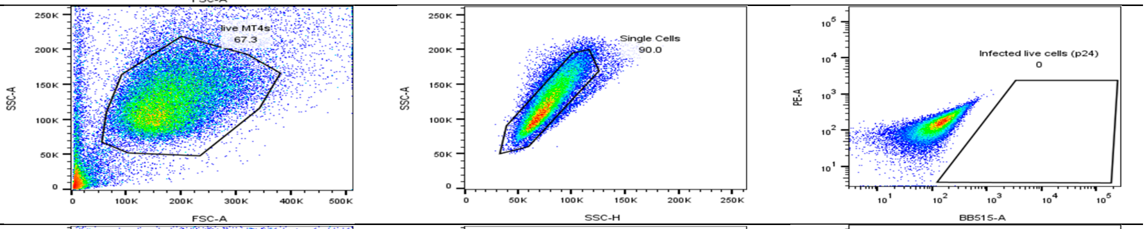

HIV (1:10) + 500 µg/mL

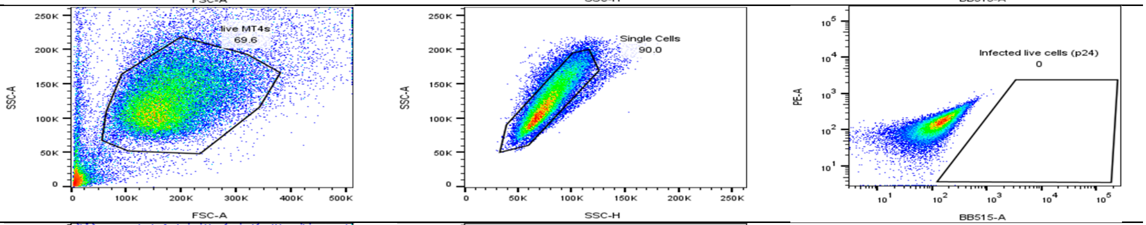

HIV (1:10) + 250 µg/mL

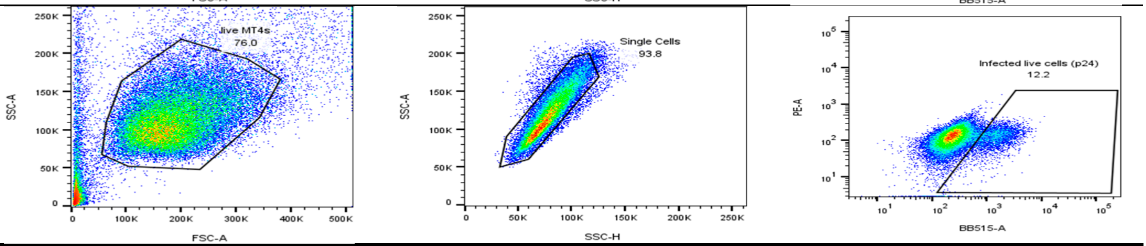

Table S3. Post hoc pairwise comparisons (FDR-adjusted *p*-values) of cytokines and chemokines secretion that were significantly changed by different treatments in the prophylactic treatment strategy. The experiments were conducted in triplicate and repeated in two independent experiments (N = 6). Statistical significance is indicated by asterisks as follows: $p < 0.05$ *, $p < 0.01$ **, and $p < 0.001$ ***.

Treatments	IL-1α	IL-10	MIP-1β
Uninfected vs. HIV	0.676	0.052	0.291
Uninfected vs. HIV + PN685	0.606	0.970	0.517
Uninfected vs. HIV + PN500	0.128	0.480	0.062
Uninfected vs. HIV + PN250	0.128	0.140	0.877
Uninfected vs. Ralt	0.139	0.970	0.250
HIV vs. HIV + PN685	0.339	0.052	0.624
HIV vs. HIV + PN500	0.058	0.198	0.445
HIV vs. HIV + PN250	0.058	0.614	0.250
HIV vs. Ralt	0.058	0.052	0.878
HIV + PN685 vs. HIV + PN 500	0.345	0.480	0.250
HIV + PN685 vs. HIV + PN 250	0.339	0.140	0.445
HIV + PN685 vs. Ralt	0.377	0.970	0.555
HIV + PN500 vs. HIV + PN 250	0.939	0.480	0.062
HIV + PN500 vs. Ralt	0.939	0.517	0.480
HIV + PN250 vs. Ralt	0.939	0.152	0.250

Table S4. Effect of pre-treatment of MT4 cells with PN for 8 h, followed by HIV infection. Uninfected, unstained, and uninfected stained cells served as negative controls. Infected, stained, and untreated cells, and raltegravir-treated infected cells served as the experimental samples.

Sample	MT4 Cells	Single Cells	Live Infected Cells
Uninfected (Unstained)	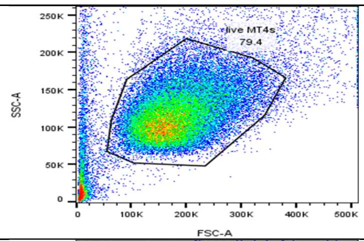	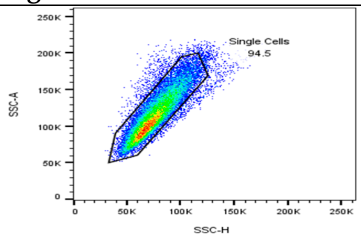	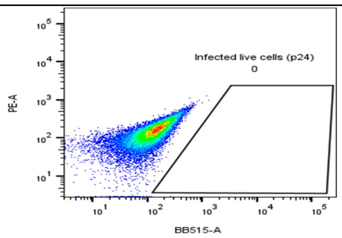
Uninfected (stained)	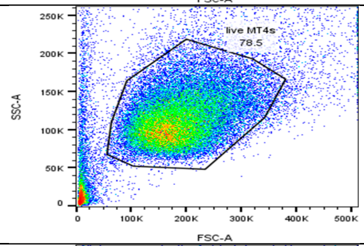	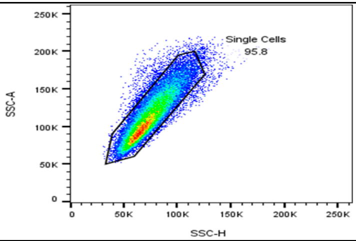	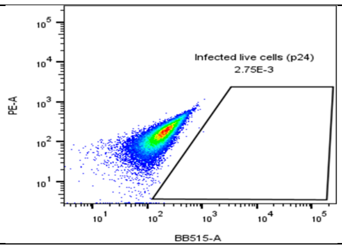
HIV (1:10)	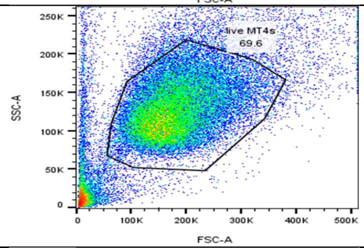	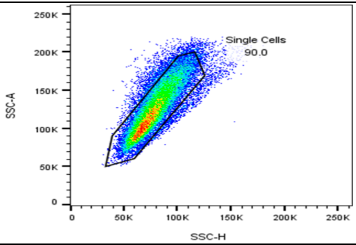	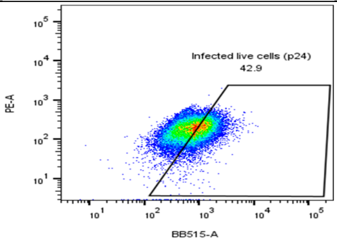
Raltergravir (7.8 µg/mL)	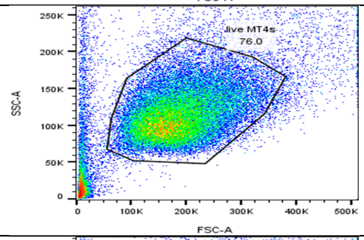	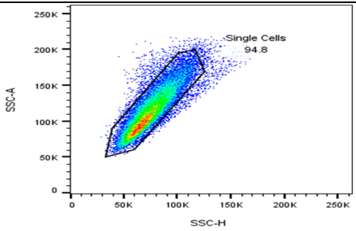	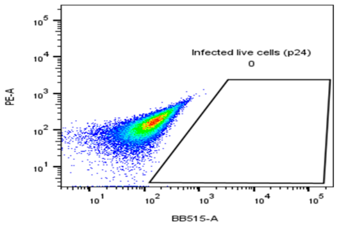
HIV (1:10) + PN 685 µg/mL	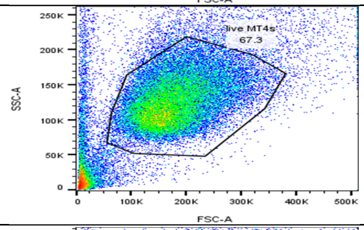	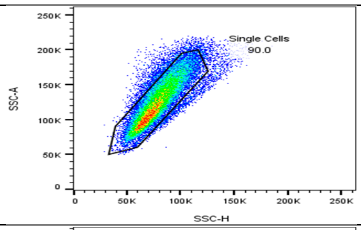	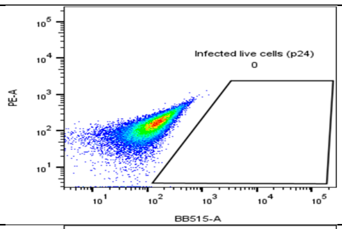
HIV (1:10) + 500 µg/mL	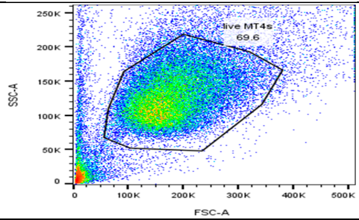	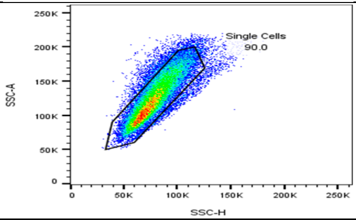	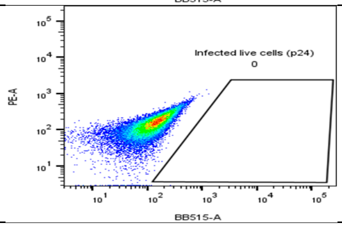
HIV (1:10) + 250 µg/mL	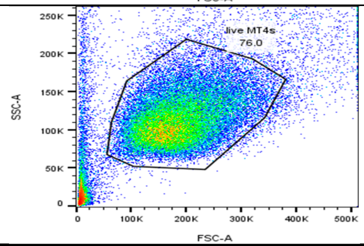	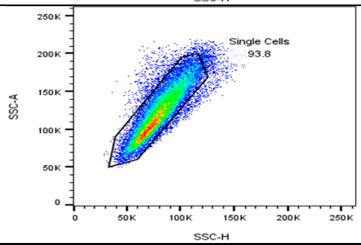	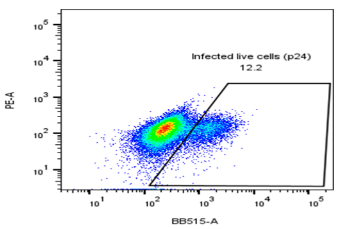

Table S5. Post hoc pairwise comparisons (FDR-adjusted *p*-values) of cytokines and chemokines secretion that were significantly changed by different treatments in the post-infection treatment strategy. The experiments were conducted in triplicates and repeated in two independent experiments (N = 6). Statistical significance is indicated by asterisks where $p < 0.05$ *, $p < 0.01$ **, and $p < 0.001$ ***.

Treatments	IL-1α	IL-6	IL-10
Untreated vs. HIV	0.269	0.022 *	0.120
Untreated vs. HIV + PN685	0.342	0.605	0.746
Untreated vs. HIV + PN500	1.000	0.228	0.909
Untreated vs. HIV + PN250	0.269	0.277	0.250
Untreated vs. Ralt	0.909	0.414	0.354
HIV vs. HIV + PN685	0.049 *	0.119	0.217
HIV vs. HIV + PN500	0.269	0.414	0.121
HIV vs. HIV + PN250	0.029 *	0.339	0.614
HIV vs. Ralt	0.300	0.228	0.009 *
HIV + PN685 vs. HIV + PN 500	0.342	0.445	0.783
HIV + PN685 vs. HIV + PN 250	0.877	0.537	0.364
HIV + PN685 vs. Ralt	0.300	0.692	0.250
HIV + PN500 vs. HIV + PN 250	0.269	0.848	0.274
HIV + PN500 vs. Ralt	0.909	0.644	0.322
HIV + PN250 vs. Ralt	0.269	0.692	0.044 *

Table S6. Effect of PN concentrations on the post-infection treatment strategy. Uninfected, unstained, and uninfected stained cells served as negative controls. Infected, stained, and untreated cells and raltegravir-treated infected cells served as the experimental samples.

Sample	MT4 Cells	Single Cells	Live Infected Cells
Uninfected (Unstained)	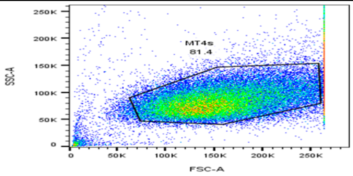	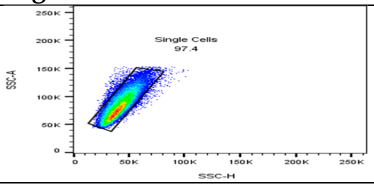	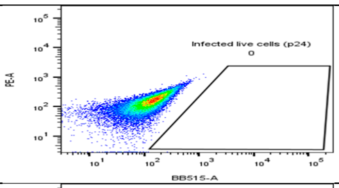
Uninfected (stained)	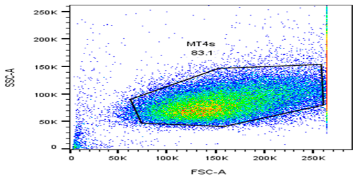	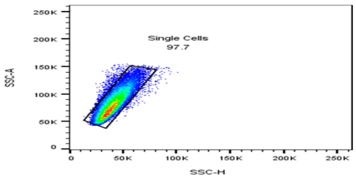	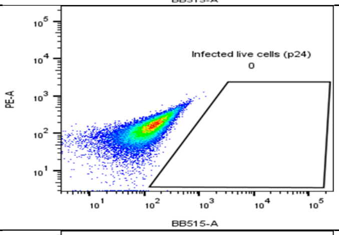
HIV (1:10)	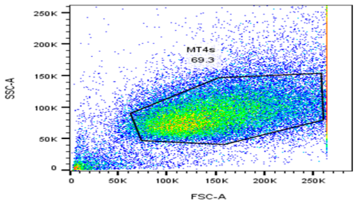	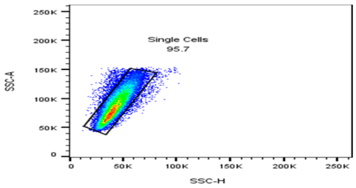	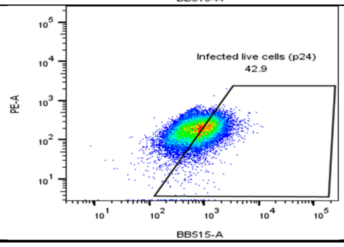

Raltergravir (7.8 µg/mL)

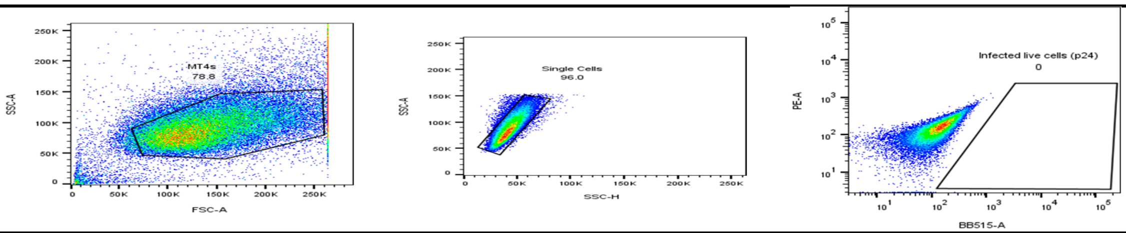

HIV (1:10) + PN 685 µg/mL

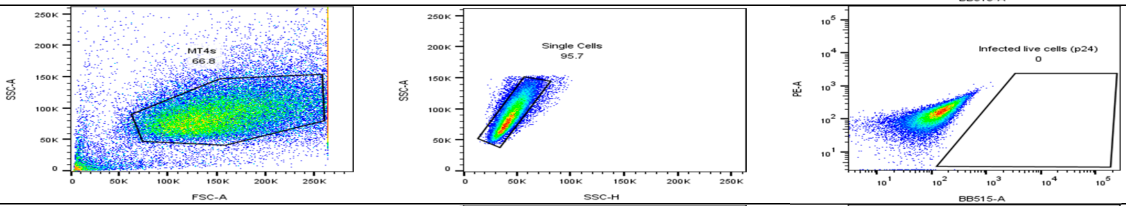

HIV (1:10) + 500 µg/mL

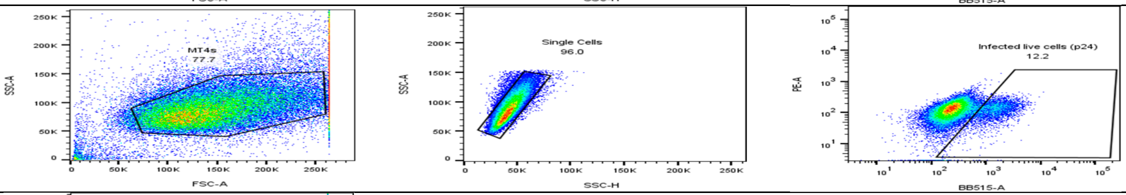

HIV (1:10) + 250 µg/mL

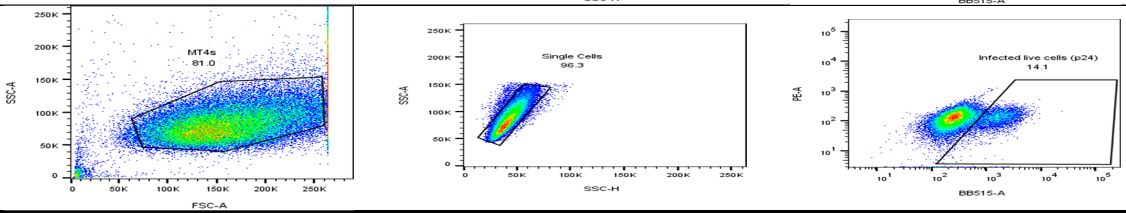

Supplementary Data S2

Table S7. Illustrated mean (SD), *p* values, and effect size of cytokine readings.

Approach 1: Neutralization strategy								
Cytokine	Untreated	HIV	HIV + PN (685 μg/mL)	HIV + PN (500 μg/mL)	HIV + PN (250 μg/mL)	Raltegravir (7.8 μg/mL)	Effect Size (ε^2)	*p*-value
GM-CSF	1.13 ± 0.0	1.13 ± 0.0	1.13 ± 0.0	1.13 ± 0.0	1.13 ± 0.0	1.13 ± 0.0	N/A	N/A
IL-1α	0.47 ± 0.06	0.33 ± 0.08	0.75 ± 0.09	0.47 ± 0.11	0.67 ± 0.08	0.33 ± 0.09	0.853	p = 0.0133
IL-1β	4.0 ± 0.0	4.0 ± 0.0	4.0 ± 0.0	4.0 ± 0.0	4.0 ± 0.0	4.0 ± 0.0	N/A	N/A
IL-7	1.8 ± 0.0	1.8 ± 0.0	1.8 ± 0.0	1.8 ± 0.0	1.8 ± 0.0	1.8 ± 0.0	N/A	N/A
IL-6	0.58 ± 0.04	0.09 ± 0.03	0.19 ± 0.01	0.28 ± 0.03	0.28 ± 0.1	0.23 ± 0.03	0.863	p = 0.0123
IL-8	2.53 ± 0.28	9.21 ± 0.91	6.73 ± 1.36	5.66 ± 1.97	8.73 ± 0.1	2.85 ± 0.87	0.853	p = 0.0132
IL-10	1.57 ± 0.49	0.38 ± 0.1	1.57 ± 0.28	1.33 ± 0.21	0.75 ± 0.09	4.24 ± 0.62	0.883	p = 0.0105
IP-10	21.33 ± 1.77	24.38 ± 4.4	9.74 ± 3.37	12.66 ± 4.76	19.51 ± 6.57	14.35 ± 3.68	0.693	p = 0.0386
MCP-1	5.44 ± 0.67	6.18 ± 1.06	3.15 ± 1.09	3.19 ± 1.06	3.89 ± 1.33	6.22 ± 0.62	0.813	p = 0.0175
MIP-1α	3153.11 ± 126.15	3776.7 ± 501.6	3249.41 ± 1075.14	2697.79 ± 888.14	3270.62 ± 1123.95	5014.48 ± 1540.81	0.543	p = 0.1001
MIP-1β	20.97 ± 2.54	27.69 ± 2.99	25.57 ± 10.75	25.01 ± 8.83	30.95 ± 13.57	32.88 ± 9.22	0.243	p = 0.5288
TNF-α	51.32 ± 2.89	54.63 ± 5.92	41.33 ± 14.61	40.07 ± 15.23	51.51 ± 17.86	56.03 ± 11.7	0.273	p = 0.4602
Approach 2: Prophylactic strategy								
Cytokine	Untreated	HIV	HIV + PN (685 μg/mL)	HIV + PN (500 μg/mL)	HIV + PN (250 μg/mL)	Raltegravir (7.8 μg/mL)	Effect Size (ε^2)	*p*-value
GM-CSF	1.13 ± 0.0	1.13 ± 0.0	1.13 ± 0.0	1.13 ± 0.0	1.13 ± 0.0	1.13 ± 0.0	N/A	N/A
IL-1α	0.9 ± 0.46	1.33 ± 0.07	0.33 ± 0.09	0.19 ± 0.04	0.19 ± 0.1	0.2 ± 0.06	0.793	p = 0.0190
IL-1β	4.0 ± 0.0	4.0 ± 0.0	4.0 ± 0.0	4.0 ± 0.0	4.0 ± 0.0	4.0 ± 0.0	N/A	N/A
IL-7	1.8 ± 0.0	1.8 ± 0.0	1.8 ± 0.0	1.8 ± 0.0	1.8 ± 0.0	1.8 ± 0.0	N/A	N/A
IL-6	0.09 ± 0.04	0.58 ± 0.05	0.19 ± 0.08	0.28 ± 0.12	0.28 ± 0.25	0.74 ± 0.54	0.563	p = 0.0896
IL-8	4.64 ± 3.9	9.4 ± 0.98	7.32 ± 3.95	11.31 ± 5.28	6.53 ± 3.15	6.27 ± 2.9	0.313	p = 0.3853
IL-10	1.57 ± 0.25	0.38 ± 0.15	1.57 ± 0.08	1.33 ± 0.09	0.75 ± 0.09	1.65 ± 0.45	0.783	p = 0.0208
IP-10	27.65 ± 3.55	25.57 ± 2.44	28.46 ± 8.45	31.82 ± 6.79	28.89 ± 11.21	26.24 ± 8.63	0.102	p = 0.8916
MCP-1	5.98 ± 0.78	5.3 ± 1.37	4.79 ± 0.79	5.48 ± 0.38	5.03 ± 2.25	5.68 ± 0.63	0.163	p = 0.7477
MIP-1α	3389.03 ± 272.36	4622.11 ± 1013.14	4219.16 ± 1362.16	5654.65 ± 884.87	3457.34 ± 1350.96	3719.87 ± 229.2	0.473	p = 0.1605
MIP-1β	23.63 ± 2.18	29.96 ± 6.61	27.24 ± 8.94	45.17 ± 9.19	20.5 ± 7.25	28.66 ± 1.12	0.693	p = 0.0394

TNF-α	52.49 ± 2.13	54.54 ± 7.11	56.94 ± 4.39	70.75 ± 21.8	44.43 ± 15.48	55.28 ± 2.78	0.433	p = 0.1961
Approach 3: Treatment strategy								
Cytokine	Untreated	HIV	HIV + PN (685 μg/mL)	HIV + PN (500 μg/mL)	HIV + PN (250 μg/mL)	Raltegravir (7.8 μg/mL)	Effect Size (ε^2)	p-value
GM-CSF	1.13 ± 0.0	1.13 ± 0.0	1.13 ± 0.0	1.13 ± 0.0	1.13 ± 0.0	1.13 ± 0.0	N/A	N/A
IL-1α	0.33 ± 0.13	1.33 ± 0.15	0.2 ± 0.06	0.33 ± 0.12	0.18 ± 0.06	0.33 ± 0.1	0.713	p = 0.0340
IL-1β	4.0 ± 0.0	4.0 ± 0.0	4.0 ± 0.0	4.0 ± 0.0	4.0 ± 0.0	4.0 ± 0.0	N/A	N/A
IL-7	1.8 ± 0.0	1.8 ± 0.0	1.8 ± 0.0	1.8 ± 0.0	1.8 ± 0.0	1.8 ± 0.0	N/A	N/A
IL-6	0.09 ± 0.04	0.58 ± 0.08	0.19 ± 0.16	0.28 ± 0.06	0.28 ± 0.15	0.19 ± 0.06	0.693	p = 0.0395
IL-8	9.03 ± 0.37	8.15 ± 1.33	9.69 ± 2.89	5.92 ± 2.95	9.03 ± 1.87	8.5 ± 1.44	0.283	p = 0.4385
IL-10	1.57 ± 0.51	0.45 ± 0.16	1.31 ± 0.29	1.52 ± 0.25	0.75 ± 0.11	2.33 ± 0.22	0.883	p = 0.0107
IP-10	18.63 ± 9.47	21.3 ± 5.94	16.68 ± 5.55	19.02 ± 6.48	25.1 ± 9.17	16.66 ± 0.15	0.132	p = 0.8187
MCP-1	8.23 ± 0.72	4.78 ± 1.83	3.2 ± 1.31	4.32 ± 1.72	4.49 ± 1.33	3.91 ± 0.68	0.513	p = 0.2501
MIP-1α	4004.47 ± 409.29	4104.8 ± 933.34	4470.99 ± 1224.68	3633.39 ± 107.76	3987.71 ± 1128.85	3806.18 ± 595.2	0.102	p = 0.8795
MIP-1β	31.62 ± 8.51	24.25 ± 2.5	33.21 ± 6.87	28.14 ± 2.26	27.26 ± 5.11	29.77 ± 1.45	0.363	p = 0.2971
TNF-α	58.28 ± 13.38	50.27 ± 9.4	61.18 ± 17.61	56.75 ± 1.17	53.84 ± 11.88	52.93 ± 0.06	0.143	p = 0.7836

Table S8. The median and interquartile range (IQR) of the different cytokines and chemokines.

**Cytokine**	**Neutralization Strategy**	**Prophylactic Strategy**	**Post-Infection Treatment Strategy**
	**Median (IQR)**	**Median (IQR)**	**Median (IQR)**
**IL-6**	0.255 (0.190, 0.280)	0.210 (0.150, 0.420)	0.235 (0.190, 0.280)
**IL-1α**	0.470 (0.330, 0.670)	0.265 (0.190, 0.900)	0.330 (0.200, 0.330)
**GM-CS**	1.125 (1.125, 1.125)	1.125 (1.125, 1.125)	1.125 (1.125, 1.125)
**IL-10**	1.450 (0.750, 1.570)	1.335 (0.680, 1.580)	1.365 (1.000, 1.640)
**IL-7**	1.805 (1.805, 1.805)	1.805 (1.805, 1.805)	1.805 (1.805, 1.805)
**IL-1β**	4.000 (4.000, 4.000)	4.000 (4.000, 4.000)	4.000 (4.000, 4.000)
**MCP-1**	4.663 (3.187, 6.183)	5.380 (4.330, 5.700)	4.010 (3.520, 6.935)
**IL-8**	6.1967 (2.8500, 8.7300)	8.385 (3.365, 9.545)	8.730 (7.370, 9.330)
**IP-10**	16.932 (12.657, 21.333)	27.465 (24.305, 35.365)	17.105 (15.330, 25.415)
**MIP-1β**	26.630 (25.007, 30.947)	27.385 (25.090, 32.795)	28.355 (25.885, 31.460)
**TNF-α**	51.413 (41.330, 54.630)	54.415 (52.230, 57.275)	55.295 (52.933, 58.280)
**MIP-1α**	3260.017 (3153.110, 3776.697)	3761.295 (3454.385, 4495.755)	3663.485 (3534.490, 4211.850)

Table S9. The selective index of Product Nkabinde.

	**IC50**	**EC50**	**SI**
Strategy 1	2210	239.2	9.26
Strategy 2	2210	239.2	9.26
Strategy 3	2210	210	10.55

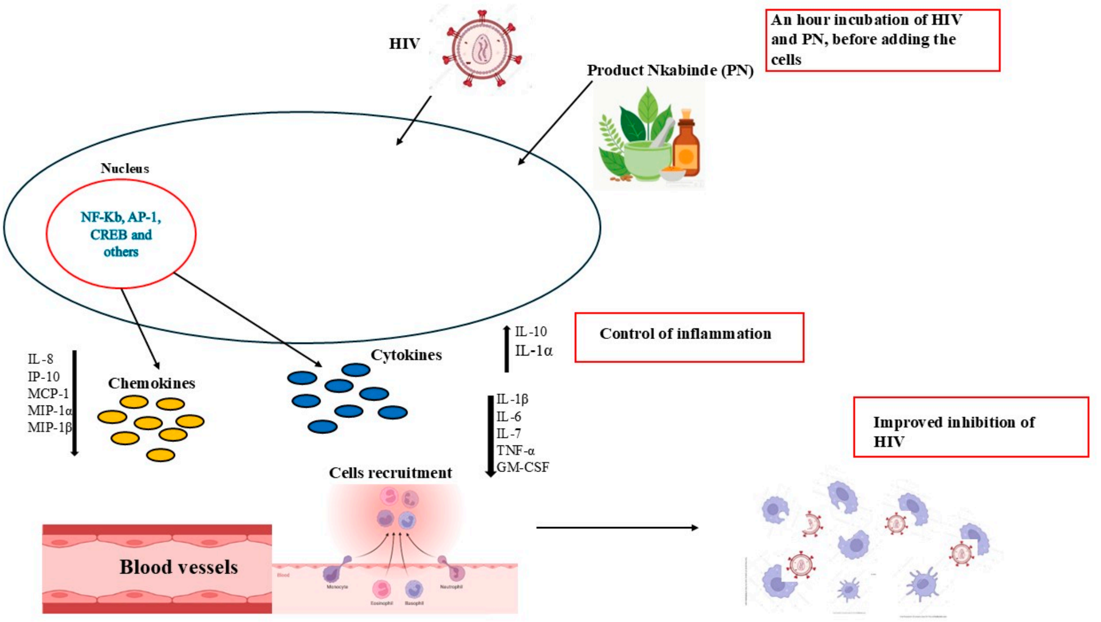

Supplementary Figure S1. PN mechanisms of immunomodulation in the neutralization strategy.

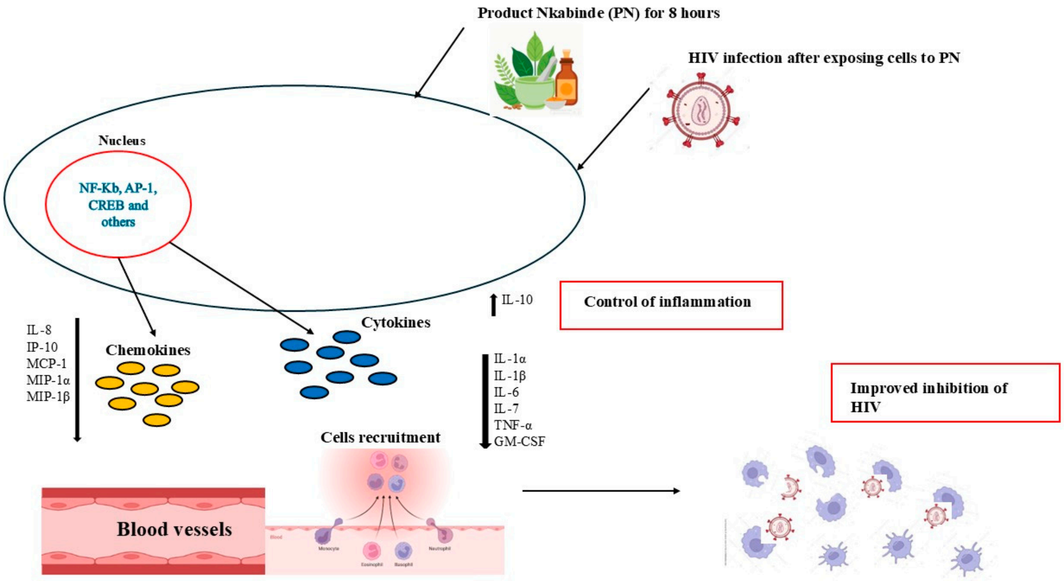

Supplementary Figure S2. PN mechanisms of immunomodulation in the prophylactic strategy.

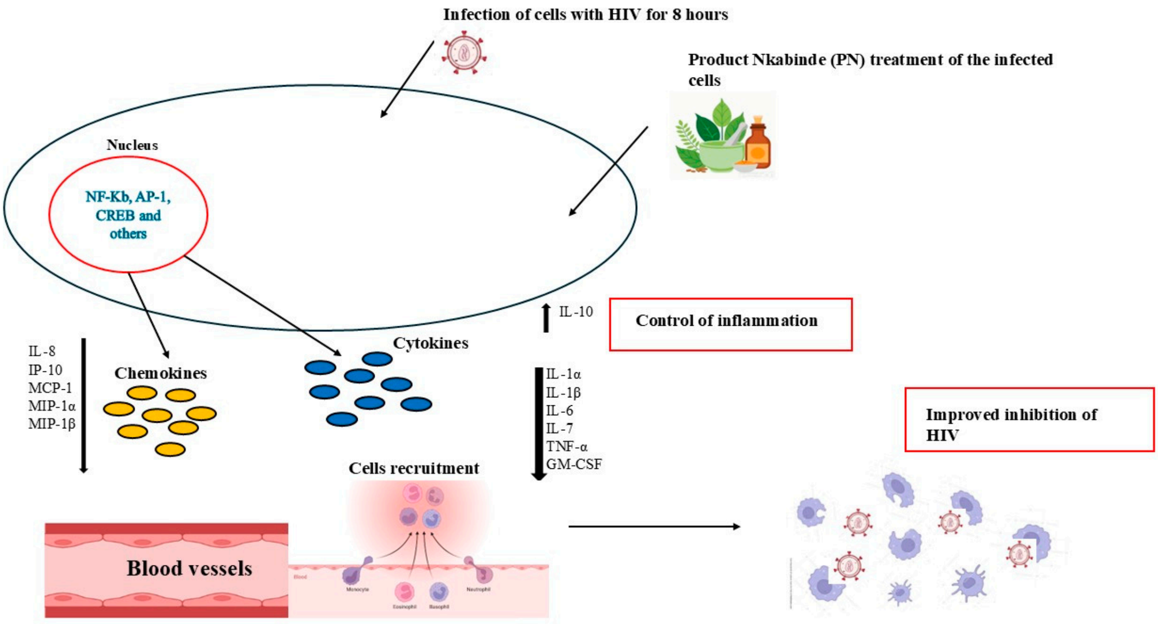

Supplementary Figure S3. PN mechanisms of immunomodulation in the treatment strategy.
